# Co-Administration of Trans-Resveratrol and L-Carnitine with a High-Fat and High-Carbohydrate Diet Modulates Liver Transcriptome in Obesity-Resistant DBA/2J Mice

**DOI:** 10.3390/ijms27177808

**Published:** 2026-08-31

**Authors:** Ivan V. Gmoshinski, Nikita V. Trusov, Vladimir A. Shipelin, Dmitriy B. Nikityuk

**Affiliations:** 1 Federal Research Centre of Nutrition and Biotechnology, 109240 Moscow, Russianikkitosu@yandex.ru (N.V.T.);; 2 Lopukhin Federal Research and Clinical Center of Physical-Chemical Medicine of Federal Medical Biological Agency, 119435 Moscow, Russia; 3 Department of Operative Surgery and Topographic Anatomy, I.M. Sechenov First Moscow State Medical University, 119435 Moscow, Russia; 4 Department of Ecology and Food Safety, Institute of Ecology, Peoples’ Friendship University of Russia Named After Patrice Lumumba, 117198 Moscow, Russia

**Keywords:** transcriptome, gene expression, liver, mice, obesity, fructose, fat, resveratrol, l-carnitine, obesity resistance

## Abstract

This study examined the effects of the trans-resveratrol (Res) and l-carnitine (l-Car) complex (RC) on mice genetically resistant to obesity. Male DBA/2J mice received low (RCl, 25/300 mg/kg BW) or high (RCh, 50/600 mg/kg BW) doses of the RC for 64 days in a control diet (CD) or a high-fat-high-carbohydrate diet (HFCD). Differential expression (DE) of genes in the liver was analyzed using DNA microarrays. HFCD alone altered 471 genes (1.7%) versus CD. In CD-fed mice, RCl and RCh affected 170 (0.6%) and 321 (1.2%) genes, respectively. In HFCD-fed mice, RCl and RCh affected 109 (0.4%) and 223 (0.8%) genes. Opposite DE changes between HFCD and RC + HFCD occurred in immune recognition and fat-storage genes (*Ccl24*, *Fabp7*, *Cd74*, *H2-Ab1*, *Srebf1*, etc.). Uniform responses to HFCD and RC + CD were seen in *Ppard*, *Irs-1*, *Tsku*, *Cyp26b1*, *Il1r1*, *Onecut1*, *Per1*, *Nlrd2*, *Rgs16*, *Grem2*, *Klf9*, and *Lpin1*. RC effects were consistent with liver morphology. Key RC-targeted pathways included retinoid metabolism, PPAR signaling, and antigen presentation. In HFCD-fed mice, additional pathways were steroid biosynthesis, oxylipin metabolism, and Jak-STAT signaling. DBA/2J mice may exhibit an innate compensatory response involving PPAR signaling, *Srebf1*, and *Socs2*, and the contribution of polymorphisms in these genes merits separate investigation in future studies of obesity resistance.

## 1. Introduction

The use of bioactive food compounds (BACs) in the dietary treatment of alimentary obesity and metabolic syndrome is considered a promising adjunct to the traditionally used calorie-reduced diets in combination with dosed physical activity [1,2]. The supposed beneficial therapeutic effect of BACs is associated with their effect on catabolic processes, having the ability to reduce cravings for sweet and fatty high-calorie foods and stimulating physical activity. Altogether, the use of BACs should lead to an increase in the effectiveness and durability of the main dietary treatment and better fixation of its results, including the patient’s ability to control eating behavior after the completion of the dietary therapy course [2,3]. As one of the possible approaches, it is worth noting to use complexes of BACs with various targets of metabolic action, for example, l-carnitine (l-Car) and trans-resveratrol (Res), as a part of food supplements or enriched dietary products. The first of these substances, which is responsible for the transport of fatty acids into mitochondria, can reduce the levels of free fatty acids in tissues, thereby affecting the lipogenesis gene expression. The second substance exhibits mainly an anti-inflammatory effect mediated by an epigenetic mechanism with the participation of the sirtuins system [4,5,6]. In mice studies with high-fat-diet-induced disorders, Res improved hepatic glucose metabolism through AMPK-related signaling, reduced liver steatosis and insulin resistance, and attenuated high-fat-diet-induced hepatic lipid homeostasis disorder by altering hepatic RNA methylation and PPARα-related regulation [7,8,9]. At the same time, studies of L-Car in HFD-fed mice showed reductions in body weight gain, visceral adiposity, glucose intolerance, hyperglycemia, HOMA-IR, hyperlipemia, hypertension, and inflammatory cytokines, indicating broad metabolic effects exhibited, apparently, not only by fatty acid transport [10,11]. The popularity of the combined l-Car and Res use in dietary nutrition has led to the development of several complex dietary supplements and foods for special dietary usage, with these two active components being included that are currently sold worldwide.

At the same time, the use of the above mentioned, as well as some other BACs (e.g., polyphenolic compounds-quercetin, curcumin, etc.) in the dietary therapy of obesity and related diseases cannot be considered fully scientifically substantiated. There are significant gaps in knowledge about the effector links of metabolism, which are the targets of the BACs’ action, their possible interaction, and differences in efficacy caused by the genetic polymorphisms of patients. A cycle of full-transcriptome studies conducted at the Federal Research Center of Nutrition and Biotechnology on in vivo biological models of diet-induced obesity (DIO), hyperlipidemia, and metabolic syndrome [12,13,14,15,16,17,18] made it possible to obtain consistent data on the mechanisms of action of l-Car and Res on metabolism, mediated by the expression of a complex set of genes involved in the metabolic pathways of PPAR- and JAK/STAT-signaling, biosynthesis, and transformation of steroid hormones, oxy-derivatives of polyunsaturated fatty acids PUFAs (prostanoids and oxylipins), the activity of the xenobiotic detoxification system with the participation of CYP450 superfamily monooxygenases, and conjugating enzymes. In a recent study performed on Wistar rats, we showed that disturbances in the metabolic pathways of steroid, xenobiotic, and retinoid metabolism, including the activity of CYP450 enzymes and liver-conjugating enzymes, are reversed as a result of the consumption of a complex dietary supplement containing Res and l-Car, if it is supplied to animals against the background of DIO induced by feeding with high-fat, high-carbohydrate diet (HFCD) [18]. However, also of interest is the problem of how these changes may vary according to the genotype of the organism, which determines susceptibility to or resistance against DIO. According to several previous studies, DBA/2J mice demonstrate genetically predetermined resistance to the development of DIO if compared to alternative models in rodents, including male and female Wistar rats, hereditarily obese Zucker ZF rats, leptin-free diabetic db/db mice, and DBCB tetrahybrid mice characterized by their transcriptome response profile [13,15,16,18]. Comparing C57BL/6J and DBA/2J mice showed that high-fat feeding triggers sex- and strain-dependent phenotypic, hepatic, adipose, and neurochemical responses, supporting the use of DBA/2J mice as a model for studying strain-dependent adaptation to obesogenic diets [19,20]. Therefore, this study aimed to evaluate the effect of a combined dietary supplement containing Res and L-Car on the liver transcriptome (general RNA expression profile) of DBA/2J mice, a strain that shows strain-dependent differences in susceptibility to diet-induced obesity.

## 2. Results

### 2.1. Integral and Morphological Data of Animals

**Figure 1:**
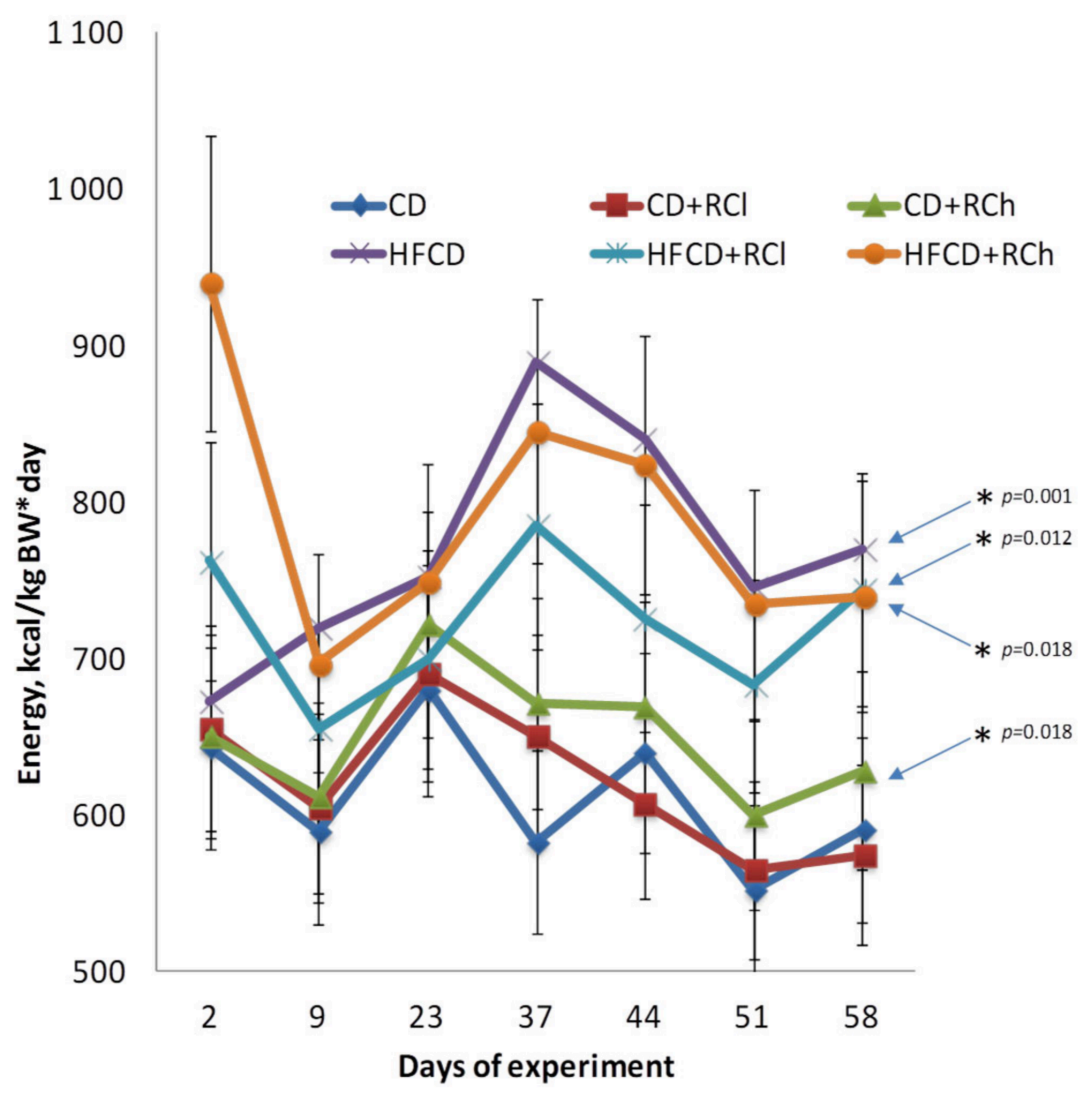
Average specific energy consumption, kcal/kg of BW per day, M ± s.e.m, in mice of six groups during the experiment. * compared to the CD group.

At the end of the feeding period, as follows from the data in Figure 1, mice of all groups fed with HFCD consumed more energy per unit of body weight compared to animals treated with CD. At the same time, the supplementation with RCh caused an increase in specific energy consumption in mice fed with CD. Against the background of the consumption of HFCD, RC did not affect the consumed energy value of the diet.

**Figure 2:**
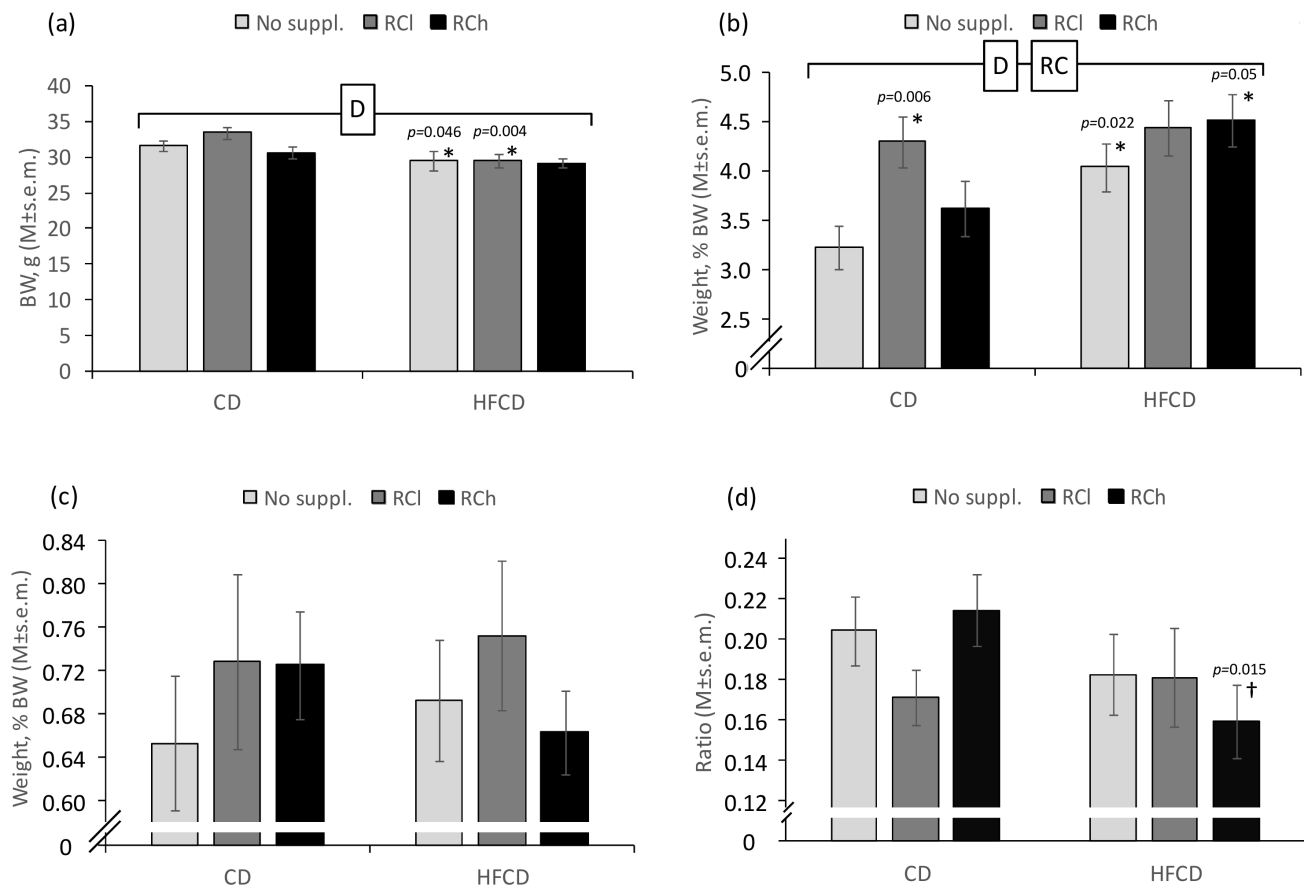
BW (**a**), white (**b**), and brown (**c**) adipose tissue and the ratio of their masses (**d**) in mice at the end of the feeding period. * compared with the CD group; † compared with the CD + RCh group. The distribution is heterogeneous (*p* < 0.05, ANOVA) by the factors “diet” (D); “dose of the supplement” (RC).

As follows from the data in Figure 2, mice that consumed HFCD did not show an increase in body weight compared to those that received CD at the end of the experiment. At the same time, the intake of HFCD corresponded to a significant increase of 71% in the mass of abdominal white adipose tissue; the consumption of RCl in mice treated with CD led to a similar effect (an increase of 87%). The amount of interscapular brown fat in mice of all groups did not differ significantly, and its proportion in relation to abdominal white fat was reduced by 43% as a result of the consumption of RCh in mice on HFCD compared to CD. Thus, DBA/2J mice in general did not demonstrate the phenotype of obesity, which is consistent with the previously proven fact of their resistance to DIO development when consuming HFCD [16].

**Figure 3:**
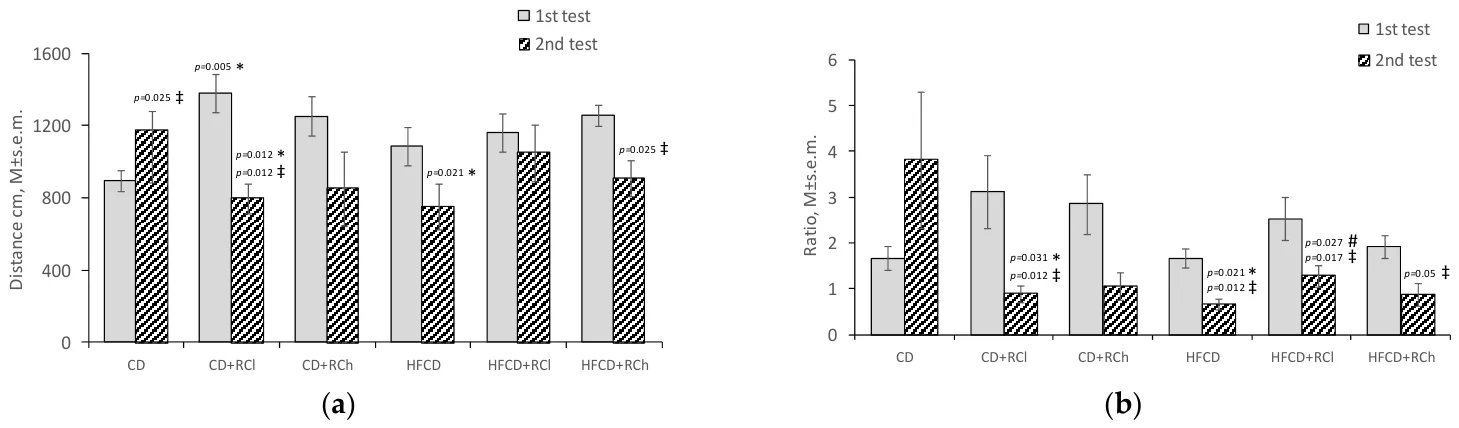
Mice testing in the EPM test: (**a**) the distance traveled in the CA, cm (M ± s.e.m.) and (**b**) the ratio of the residence times in the CA and OA, dimensionless (M ± s.e.m.). * The difference with the CD group is significant; # the difference with the HFCD group is significant; ‡ the difference between test 1 and test 2 is significant, the paired Wilcoxon test.

Testing of mice in the “Elevated plus maze” (EPM) showed (Figure 3) that the distance traveled in the closed arms (CA) of the maze significantly increased by 35% in the second test compared to the first in mice of the first group fed with CD. The consumption of RC in the composition of CD, HFCD, and their combinations with RC at least canceled this change. The locomotor activity of mice treated with HFCD and RCl as a part of CD was weakly but significantly reduced compared to the CD group by 19 and 9%, respectively. The index of anxiety-like functions measured as a ratio of the time spent in the CA to the open arms (OA) was the highest in the second test in mice treated with CD. Consumption of RC together with CD and HFCD led to a significant decrease in anxiety compared with the CD group by 82 and 91%, respectively. At the same time, anxiety increased by 95% in mice treated with RCl as part of the HFCD, compared with the HFCD group.

**Figure 4:**
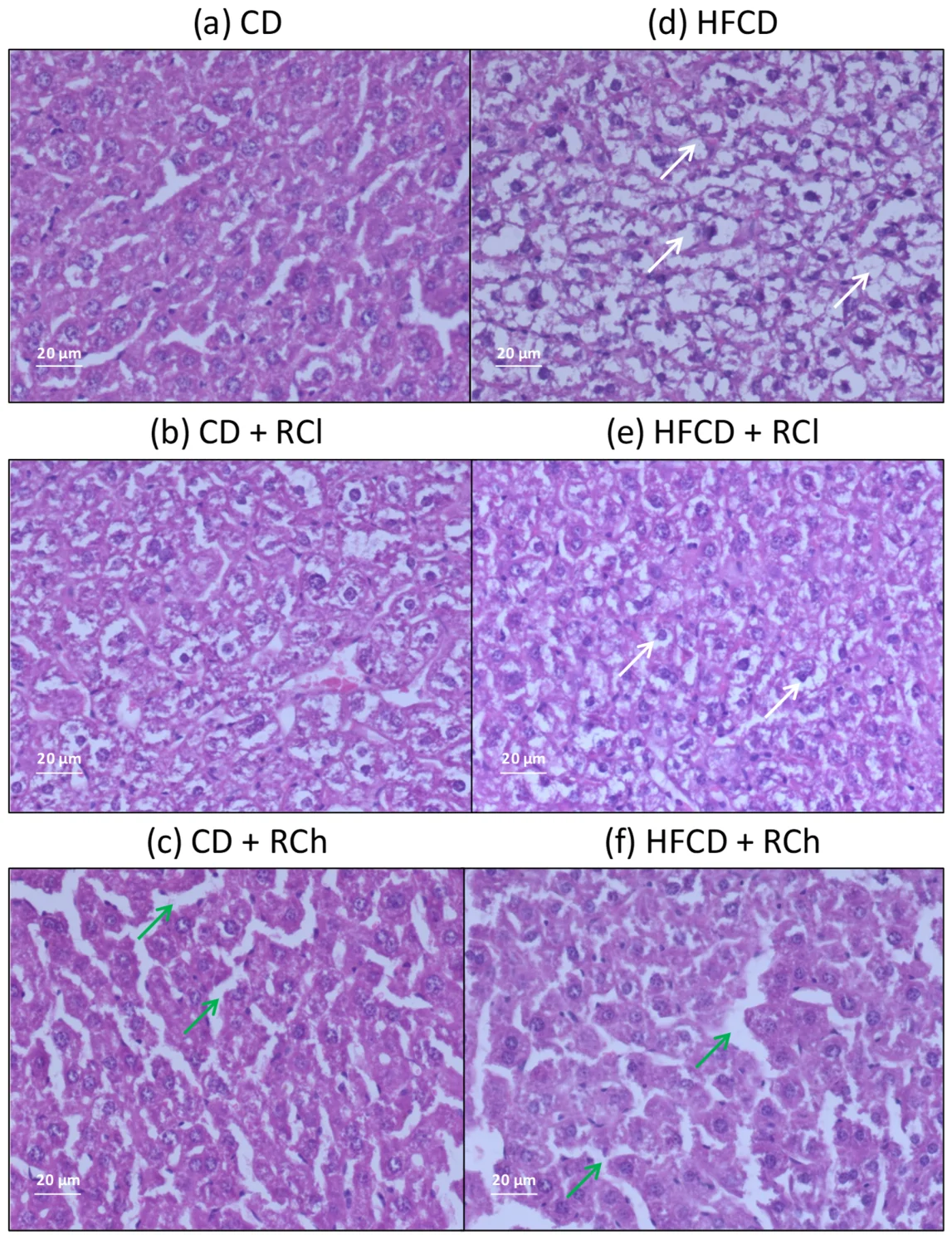
Representative photomicrographs of liver sections from mice treated with CD (**a**), CD with RCl (**b**), CD with RCh (**c**), HFCD (**d**), HFCD with RCl (**e**), and HFCD with RCh (**f**). White arrows indicate areas of hepatic steatosis, and green arrows indicate dilated bile ducts. Stained with hematoxylin-eosin, magnification ×400.

The structure of the liver (Figure 4) in mice treated with HFCD was characterized by diffuse accumulation of fat in most liver cells, including hepatocytes, which was almost not observed in mice fed with CD. Against the background of CD consumption, RCl also led to a noticeable but less pronounced accumulation of fat, but when consuming RCh, there was practically no fat in the cells. The capillaries of the intrahepatic bile ducts were dilated, which may indicate an increase in bile secretion. There were also signs of inflammation-eosinophilic infiltration, the appearance of micronuclei and binuclear cells. With the consumption of RCl and RCh as part of HFCD, a less pronounced accumulation of fat in the liver was observed than in the absence of dietary supplements.

### 2.2. Common Description of Liver Transcriptome

**Figure 5:**
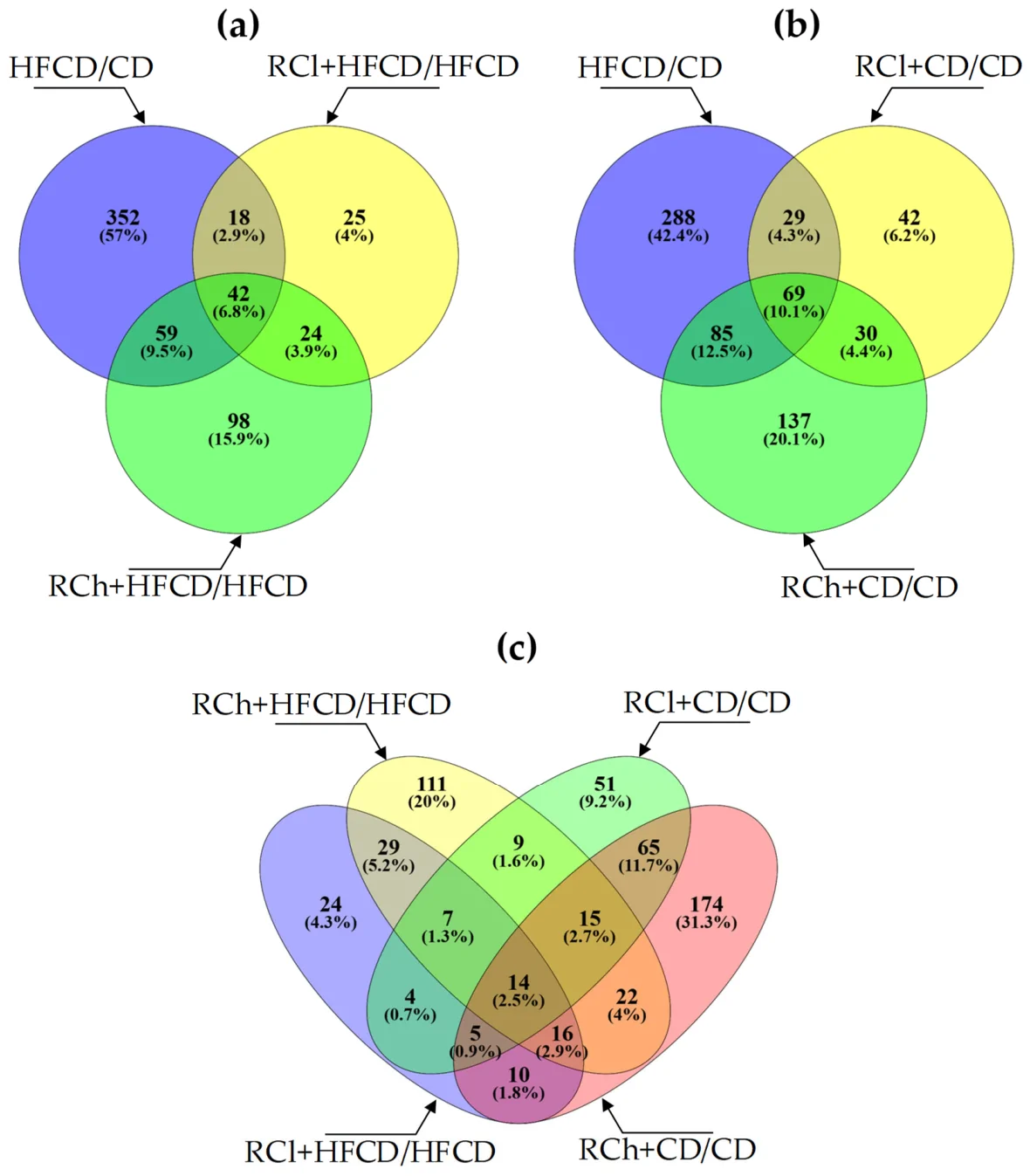
Venn diagrams demonstrating the distribution of gene DE responses to HFCD and RC as a part of HFCD (**a**), to HFCD and RC as a part of CD (**b**), and for four applications of two doses of RC in the composition of two diets (**c**).

DE of 471 genes was detected in mice treated with HFCD compared with those treated with CD, which is 1.7% of the total number of genes presented by their nucleotide sequences on the DNA microarray (Figure 5a). The number of genes that responded to DE was 170 (0.6%) when comparing mice fed with RCl as a part of CD with those receiving CD, and in RCh as a part of CD with those fed with CD, 321 genes (1.2%) (Figure 5b). In mice that received RCl as a part of HFCD, DE of 109 genes (0.4%) was noted when compared with those receiving only HFCD, and in mice fed with RCh with HFCD, 223 genes (0.8%) (Figure 5a).

The number of genes that responded with DE only to HFCD when compared with CD, but did not respond to the supplement, was 352 (1.3%) (Figure 5a). In total, 18 genes responded to the consumption of HFCD as well as consumption of RCl (but not RCh) as a part of HFCD, 24 genes responded to RCh (but not RCl) under these conditions, and 42 genes responded jointly to HFCD and both doses of RC (Figure 5a). The number of genes that jointly responded to feeding with HFCD when compared with CD and to the consumption of RCl as a part of CD was 98, 154 genes to HFCD and RCh plus CD, and 69 genes to HFCD and both doses of the supplement given as a part of CD (Figure 5b).

Of the total number of 565 genes that responded with DE to the intake of RC against the background of both CD and HFCD feeding (Figure 5c), 14 genes responded to both doses of RC in the composition of both diets, namely *Abtb2*, *Chac1*, *Cish*, *Dbp*, *Hamp*, *Hspa1a*, *Lrfn3*, *Marco*, *Mfsd2a*, *Pcsk4*, *Per1*, *Socs2*, *Tff3*, and one unidentified transcript.

Lists of genes with known functions according to the data of the US National Center for Biotechnology Information (NCBI) (http://www.ncbi.nlm.nih.gov; http://genemania.org/) that responded with DE to various doses of RC in both diets (see Figure 5c) are presented in Table 1.

**Figure 6:**
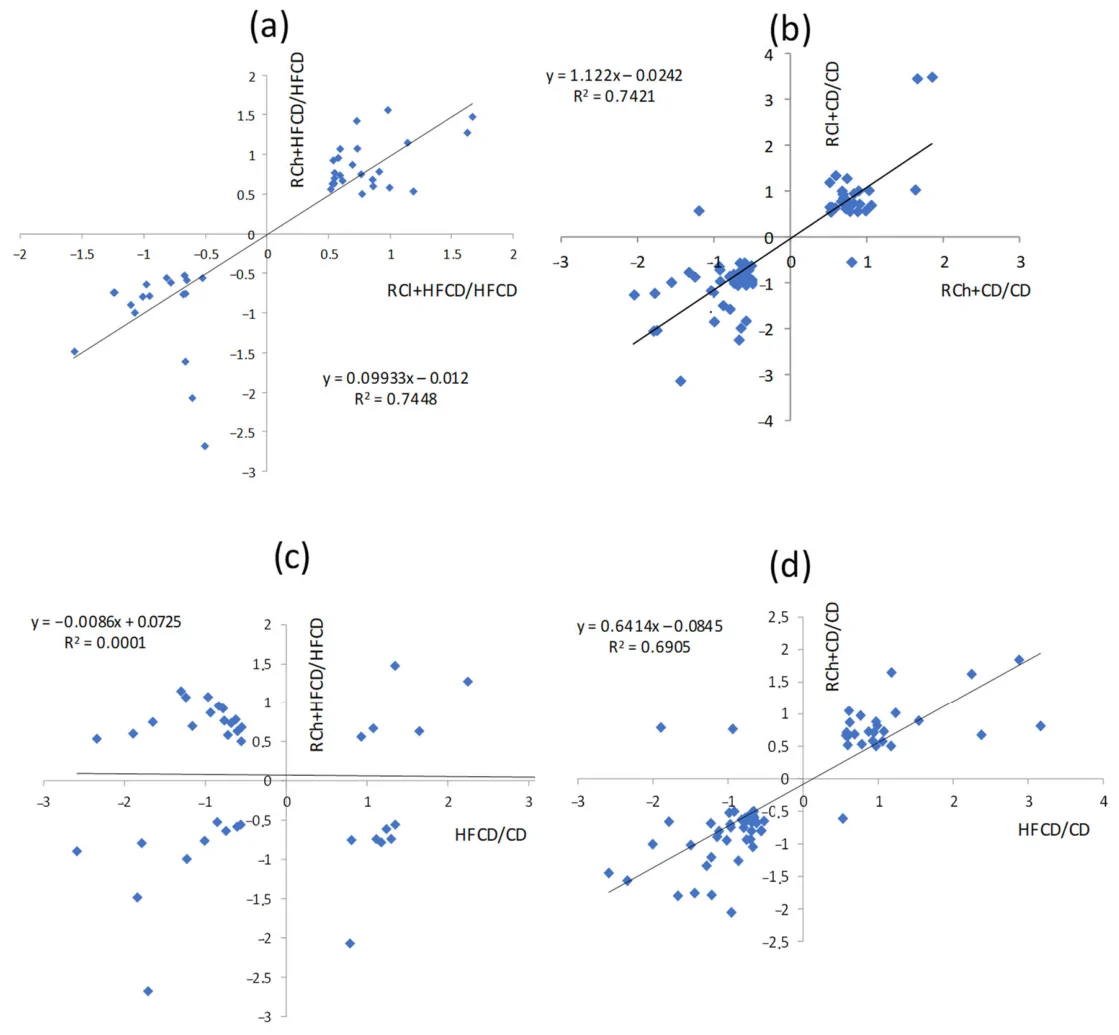
Linear regression between DEs of genes that simultaneously responded to RCl and RCh with HFCD (**a**), RCl and RCh with CD (**b**), RCh with HFCD and HFCD (**c**), and RCh with CD and HFCD (**d**). Along the axes, DE, log_2_FC.

The results of calculating the correlation coefficient and linear regression between the DE values of the genes that simultaneously responded to several of the applied dietary interventions are presented in Figure 6a,b. It can be seen that DEs of the genes that responded to both doses of the supplement (RCl and RCh) positively correlated with each when the supplement was administered as a part of HFCD (r = +0.863, *p* < 0.001) as well as a part of CD (r = +0.861, *p* < 0.001); at that, the dependence was close to linear. The dependence between the DEs of the genes that responded to RCh with HFCD and at the same time to HFCD itself was practically absent (r = +0.010, *p* > 0.05) (Figure 6c). (Similarly, there was no dependence in the case of RCl consumption.) However, the DEs of the genes that responded to RCl as a part of CD and, at the same time, to HFCD (Figure 6d) correlated positively (r = 0.837; *p* < 0.001). (A similar relationship was noted for RCh.) Thus, changes in the transcriptome caused by HFCD feeding and RC when it is consumed as a part of CD are unidirectional to a certain extent.

### 2.3. Influence of RC Supplement on Metabolic Pathways

Metabolic pathways (KEGGs) affected under the influence of applied dietary manipulations were identified with the help of bioinformatics analysis in the “R” medium. The results presented in Table 2 show that 18 metabolic pathways were significantly changed only as a result of the consumption of HFCD compared to CD. Four metabolic pathways changed only under the influence of RCl as part of the HFCD, and three for RCh as part of the HFCD. Under the influence of HFCD and RCh in the composition of HFCD, six metabolic pathways were changed simultaneously. The metabolic pathway of mmu04612 Antigen processing and presentation was significantly affected by the consumption of HFCD and both doses of RC with HFCD; mmu04630 Jak-STAT signaling pathway, under the influence of HFCD and RCh as part of CD; mmu00830 Retinol metabolism, under the influence of HFCD; and both doses of RC as part of HFCD and RCh with CD.

**Figure 7:**
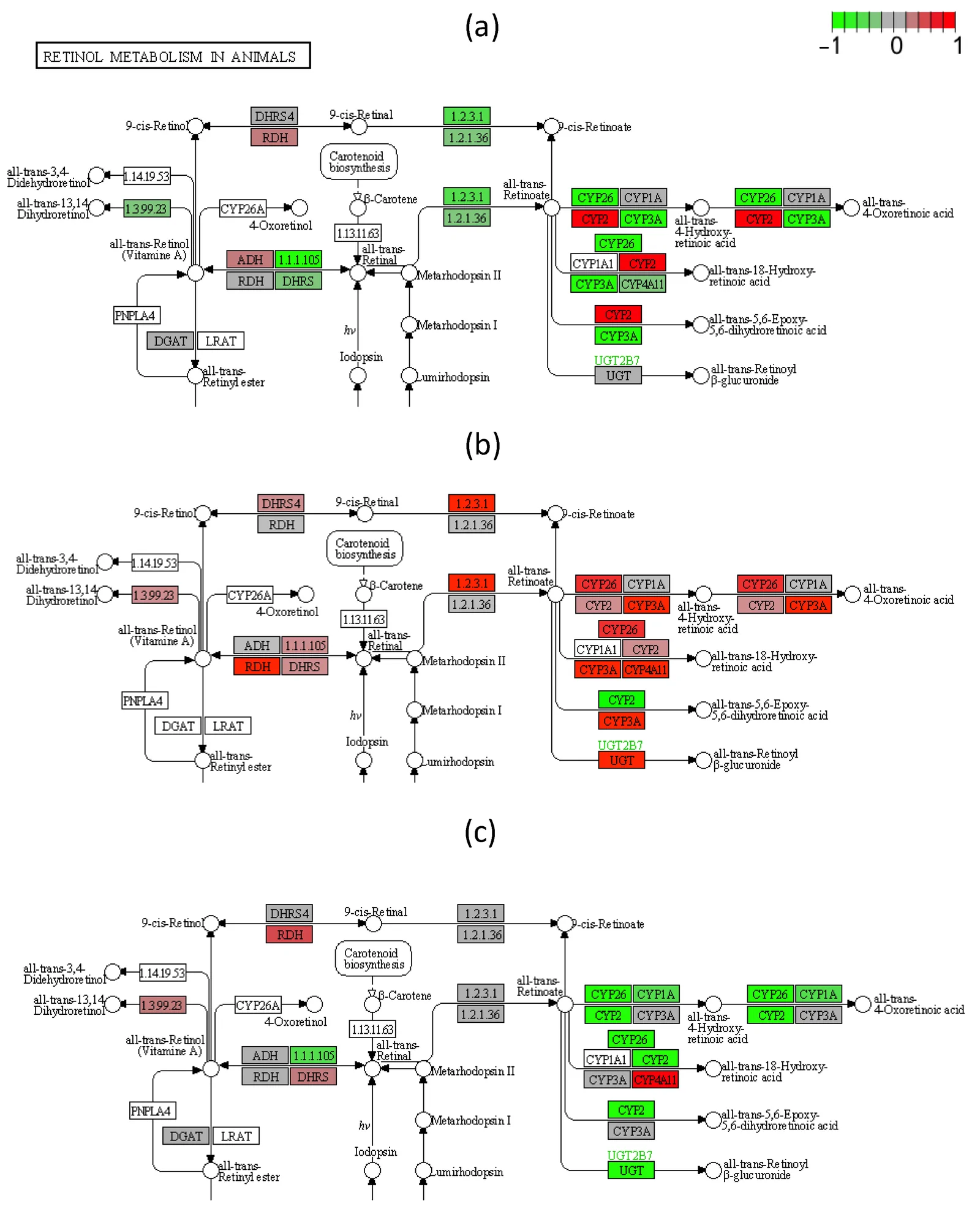
Fragments of the scheme of the metabolic pathway of Retinol metabolism, mmu00830, demonstrating changes in mice: (**a**) fed with HFCD compared with CD fed, (**b**) fed with RCh as a part of HFCD compared with HFCD fed, and (**c**) fed with RCh as a part of CD compared with CD fed. Color scale: log_2_FC, from −1 (light green, downregulation) to 1 (red, upregulation); grey indicates 0.

As can be seen from the diagram shown in Figure 7, the changes are different in the metabolic pathway of mmu00830 Retinol metabolism caused by the consumption of HFCD and by the supplementation of RC with it at different doses. Namely, in mice fed with HFCD, there is a metabolic block in the conversion of 9cis-retinal to 9cis-retinoate, whereby the first of these metabolites can accumulate. At the same time, the conversion of all-trans-retinal to all-trans-retinoate is blocked in these animals. The latter is characterized by increased metabolism mediated by CYP2 with suppression of the CYP3A, CYP4A11, and CYP26 pathways (Figure 7a). At a low dose of the supplement (RCl) administered as a part of HFCD, the metabolic pathway through CYP4A11 is restored, and on the contrary, one through CYP2 is suppressed. At a high dose of the supplement fed with HFCD (Figure 7b), the metabolic blocks for the formation of 9cis-retinoate and all-trans-retinoate are eliminated, and the pathway for converting retinol to all-trans-retinal is activated. At the same time, the process of glucuronidation of retinoic acid and its derivatives is enhanced. Upon feeding animals with RCh as part of CD (Figure 7c), the nature of changes in the metabolism of retinoids differs sharply from that which occurs when the supplement is administered as part of HFCD. Namely, retinoic acid glucuronidation is blocked, and a metabolic block occurs in the formation of all-trans-18-hydroxyretionate, all-trans-4-oxoretionate, and all-trans-5,6-epoxy-5,6-dihydroretionoate derivatives.

**Figure 8:**
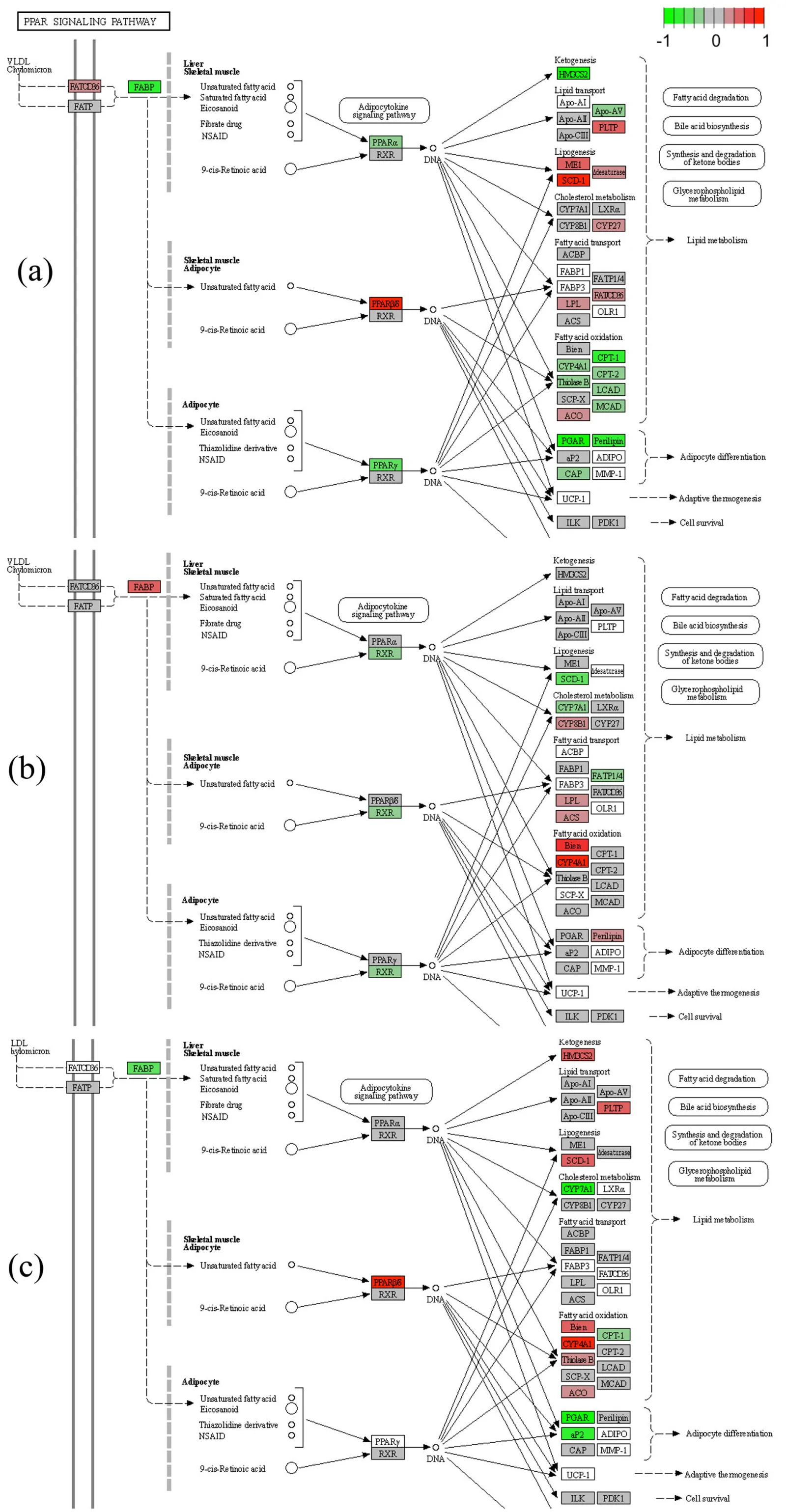
Fragments of the scheme of the metabolic pathway of PPAR signaling, mmu03320, demonstrating changes in mice: (**a**) fed with HFCD compared with CD fed, (**b**) fed with RCl as a part of HFCD compared with HFCD fed, and (**c**) fed with RCh as a part of CD compared with CD fed. Color scale: log_2_FC, from −1 (light green, downregulation) to 1 (red, upregulation); grey indicates 0.

In the metabolic pathway mmu03320 PPAR signaling pathway in mice treated with HFCD, the expression of FABP, which transports fatty acids from circulating VLDL and chylomicrons into cells, as well as the PPARα,γ signaling pathways, was suppressed upon activation of PPARβ,δ (Figure 8a). Under the influence of RCl as a part of HFCD (Figure 8b), FABP expression was restored, and negative regulation of RXR (retinoid-X-receptor) was observed. This was reflected in the observed suppression of the lipogenesis gene complex and activation of fatty acid oxidation. Inclusion of RCh into CD led (Figure 8c) to downregulation of FABP expression with accompanying upregulation of PPARβ,δ, similar to the action of HFCD by itself, resulting in the activation of lipogenic factors (*SCD-1* and *HMGCS2*).

**Figure 9:**
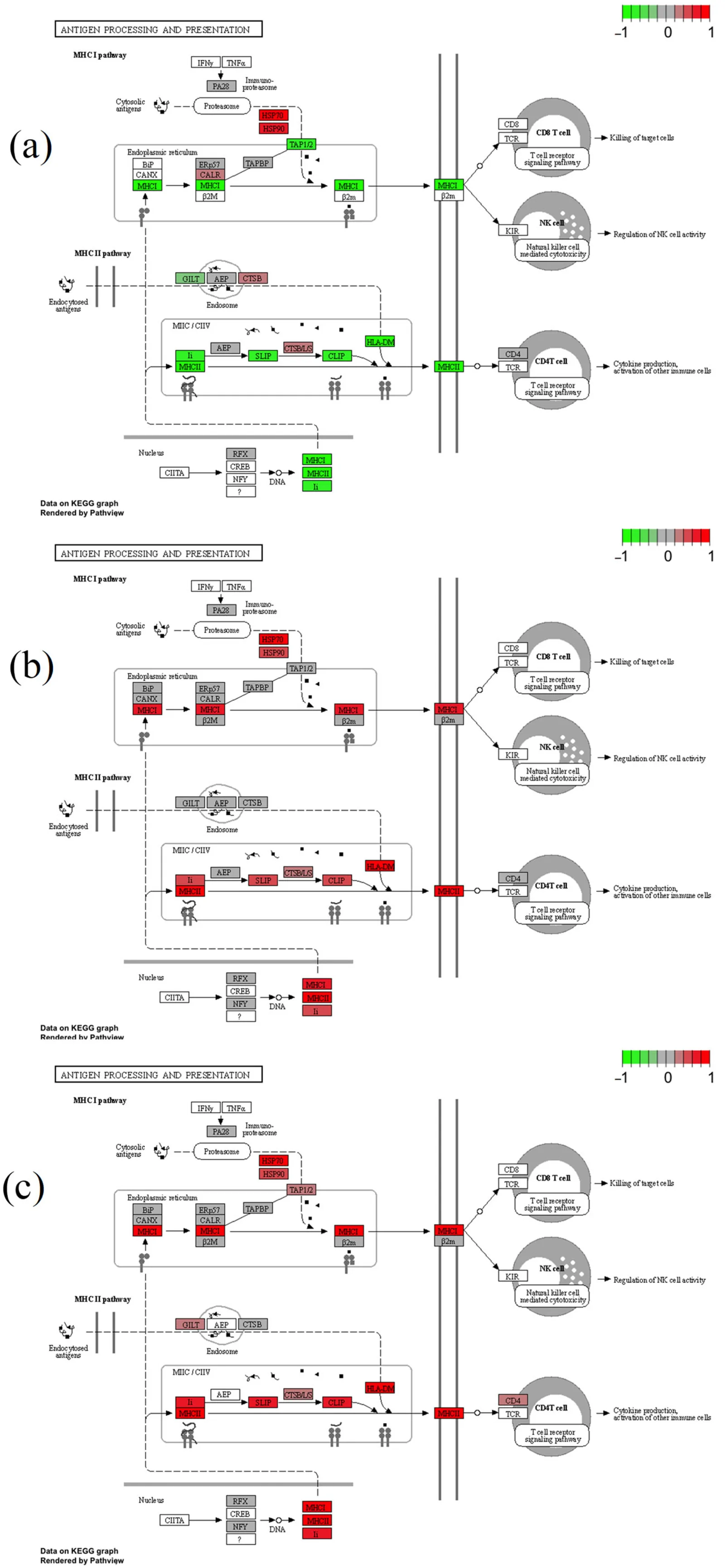
Fragments of the scheme of the “Antigen processing and presentation” metabolic pathway, mmu04612, demonstrating changes in mice: (**a**) treated with HFCD compared with CD, (**b**) RCl with HFCD compared with HFCD, and (**c**) RCh with HFCD compared with HFCD. Color scale: log_2_FC, from −1 (light green, downregulation) to 1 (red, upregulation); grey indicates 0.

In the metabolic pathway mmu04612 Antigen processing and presentation in mice fed with HFCD (Figure 9a), the expression was reduced of primary macrophage antigen recognition factors, including MHCI and MHCII, which could result in suppression of antigen presentation to all major immune cell types, including Th (CD4+), Treg (CD8+), and NK (CD158+) cells. Under the influence of RC, included in the HFCD at both doses, these disorders were partially or completely compensated (Figure 9b,c).

## 3. Discussion

In the present study, the introduction of a complex dietary supplement RC into the diet was performed in DBA/2J mice, demonstrating a relatively high resistance to the development of DIO, in contrast to alternative models in rodents, including Wistar and Zucker ZF rats, leptin-deficient mice db/db mice, and DBCB tetrahybrid mice previously used in our studies [12,15,16,18]. Although HFCD caused a statistically significant increase in abdominal white adipose tissue, the absolute magnitude of this change was small relative to total body weight, which explains why final body weight did not differ significantly. The phenotype of mice in EPM should be interpreted as an independent behavioral endpoint. In agreement with work [21] showing that HFD affects anxiety in a time and energy state-dependent manner, the DBA/2J mice in our 64-day model did not develop an anxiogenic response to HFCD consumption, which is consistent with their obesity-resistant phenotype. It is of interest to clarify the question of what changes in gene expression correspond to the revealed ambiguous effect of RC on the accumulation of fat in the liver, the development of white and brown adipose tissue in a comparative aspect in animals receiving CD and HFCD, as well as in comparison with the available data for animals of other species and lines.

It is necessary to dwell, first of all, on the group of genes characterized by a negative DE in response to HFCD and by reversal of this effect as a result of the supplementation of this diet with RC. Among them are genes functionally associated with the immune response. This is, in particular, *Ccl24*, whose expression product eotaxin-2 enhances the proliferation of eosinophils [22,23]. It is noteworthy in this regard that eosinophilic infiltration increased in the liver of mice supplied with RC. The *Fabp7* and *Ifi27l2a* genes were characterized by the same DE profile. The first of them encodes a protein that transports fatty acids and can regulate cell sensitivity to leptin [24]. The second is an interferon-induced adipokine that controls the development of white adipose tissue [25]. Negative DE in mice consuming HFCD and positive: both doses of RC as part of HFCD were also observed in the *Cd74* gene, as well as *H2-Ab1*, *H2-DMb1*, and *Igfbp6*, which are functionally related to it. The CD74 protein is required for the presentation of antigens by macrophages to T lymphocytes; its expression is associated with the development of liver steatosis in a mouse model [26]. Previously, negative DE of *Cd74* was observed by us in DBCB tetrahybrid mice that consumed HFCD [16]. *H2-Ab1* encodes the major histocompatibility complex antigen involved in the presentation of antigens by macrophages to T lymphocytes during the development of obesity-induced inflammation [27]. In our work [15], a positive DE of this gene was noted in hereditarily obese db/db mice treated with quercetin. The function of *H2-DMb1* is associated with the regulation of B lymphocytes with the participation of the IL-10 receptor. *Igfbp6* belongs to the gene family of proteins that bind insulin-like growth factor-1 (IGF-1, somatomedin) [28] and plays an active role in the regulation of glycemia and growth hormone function.

Finally, among the genes that responded with a negative DE to HFCD and a positive to RC as a part of HFCD, it should be noted *Plk3* (polo-like kinase 3), which is involved in the regulation of the cell cycle and prevents tumor growth [29], as well as *Serpina3g*, is associated with the development of sugar diabetes in leptin-free db/db mice [30], as well as *Dnaic1* (dynein), an insulin-dependent factor responsible for microtubule motility. Its positive DE was noted by us in DBCB mice in response to the addition of l-Car supplied as part of the HFCD [16].

The opposite pattern, that is, an increase in expression with the consumption of HFCD and a decrease with its supplementation with RC was noted, first of all, in *Srebf1*. It is a transcription factor that acts on the sterol-1 regulatory element (SRE1), which is included in the non-translated regions of the low-density lipoprotein receptor genes and enzymes of steroid biosynthesis [31]. Increased expression of *Srebf1* suppresses the activity of fat-storing Ito cells and fibrosis in the liver [32]. Functionally, *Srebf1* is also associated with the regulation of iron metabolism [33]. In our experiments on Wistar rats prone to the development of diet-induced obesity, the expression of *Srebf1* decreased under the influence of HFCD [18].

If, for a significant number (although not for the majority) of the DBA/2J mouse genes that responded with DE to HFCD and RC as part of HFCD, the direction of the effect was opposite, then, for the vast majority of genes with DE in response to HFCD and, at the same time, to the RC consumed as part of the CD, the sign of the DE coincided (see Figure 6). This means that both the hypercaloric diet and the supplement acted in these circumstances on the transcriptome in a similar direction. Among these genes, *Ppard* should be noted (the positive expression on HFCD and RC with CD), encoding a nuclear receptor that enhances β-oxidation of fatty acids and oxidative phosphorylation in mitochondria [34], which prevents the development of insulin resistance in liver cells under the influence of pro-inflammatory cytokines [35]. The same DE profile was characteristic of the *Irs1* insulin receptor substrate gene, one of the main components of insulin signaling that inhibits the development of diet-induced obesity [36]. Characteristically, in Sprague–Dawley rats prone to its development, the expression of *Irs1* decreased when a high-fat diet was consumed [37].

The *Tsku* gene was characterized by a unidirectional negative DE in response to HFCD and to RC as a part of the CD. It encodes a secreted liver factor, the production of which sharply increases at a high-energy expenditure [38]. An increased expression of *Tsku* was noted in liver steatosis and metabolic dysfunction-associated steatotic liver disease [39]. In experiments on animals predisposed to the development of obesity (Wistar rats and, especially, Zucker ZF rats), this gene responded to HFCD with positive DE [12].

A decrease in expression in response to HFCD and RC with CD was noted for *Cyp26b1*, which encodes a monooxygenase involved in the metabolism of retinoic acid and regulates the activity of T cells [40,41], as well as *Il1r1* (interleukin 1 receptor, CD121) [42]. IL-1RI deficiency protects against high-fat, diet-induced insulin resistance and reduces local inflammation of adipose tissue [43].

Consumption of HFCD and RC as a part of CD similarly reduced the expression of several genes associated with the regulation of the circadian rhythm. Among them are the central regulator of biological rhythms *Per1*, the expression of which, according to the literature, is suppressed with an excess of carbohydrates [44], as well as *Rgs16*, which plays the role of a circadian oscillator in the hypothalamus and liver [45], and, finally, *Nr1d2*, which is involved in maintaining a normal sleep cycle and wakefulness [46]. *Nr1d2* is involved in the regulation of eating behavior by suppressing the orexin system [47]. The same DE profile was also characteristic of the *Onecut1* gene, which is one of the candidate genes for type 2 diabetes mellitus in humans [48]. In our previous study, obese Zucker ZF rats showed a positive DE of this gene in response to HFCD [12].

A unidirectional negative DE in response to HFCD and, at the same time, to RC as a part of CD was observed in DBA/2J mice for highly specialized transcription factors associated with the development of pathology in obesity and type 2 diabetes. Among them, *Grem2* should be pointed out; its function is associated with the ability to activate JNK1 [49]. In a previous study, we showed a negative DE of this gene in C57Black/6J mice fed with HFCD and phenotypically, like DBA/2J, weakly developed of DIO at these conditions [15]. The above-said genes also included *Klf9*, a zinc finger transcription factor functionally related to *Nr1d2*, which is involved in the stimulation of adipogenesis [50] and related to *Lpin1*, phospholipase C, which generates the signaling molecule diacylglycerol [51]. In obese Zucker rats, under the action of HFCD, the expression of this gene increased [12].

Consumption of HFCD and RCh as a part of CD upregulated expression of *G6pc* gene (glucose-6-phosphatase), which is responsible for the removal of free glucose from the cell. Reduced expression of this gene in mice suppresses obesity and insulin resistance [52]. The data obtained for this gene confirm the results of a previous study, also performed on DBA/2J mice [16].

*Socs2* was a gene that had a polar opposite profile of DE response to RC against the background of consumption of CD or HFCD. It was downregulated by the consumption of HFCD as well as RC with CD, but in the case of feeding with RC as a part of HFCD, its expression increased. *Socs2* affects the glycemic response by influencing *Igf1r* (the receptor for insulin-like growth factor 1, IGF-1) and is involved in IGF-1 signal transduction [53]. *Socs2* function is also associated with *Kit*, *Lepr* (leptin receptor), and other genes of the *Socs* family. According to [54], the *Socs2* expression product is an inhibitor of cytokine signaling and an effector of the JAK/STAT pathway. The polymorphism of this gene is associated with the risk of developing type 2 diabetes mellitus [55]. According to our study [12], *Socs2* was involved in the transcriptomic response in the liver of obese Zucker rats to quercetin supplementation. The revealed changes in *Socs2* expression changes may indicate altered JAK-STAT-related metabolic signaling; however, the present study did not assess *Socs2* genetic variants; therefore, no conclusions regarding *Socs2* polymorphisms can be drawn.

For some genes belonging to the family of heat shock proteins, the response of DE in DBA/2J mice was characterized by the same direction regardless of the dietary factor (HFCD or RC as part of HFCD or CD). Thus, in all these cases, the expression of *Hspa1b* (heat shock protein 1B, a synonym for *Hsp70*) was positive. According to its function, it is a key chaperone that removes defective and damaged protein molecules in proteasomes [56], inhibits the activity of the NLRP3 inflammasome [57], and participates in reparative processes in the liver tissue [58]. Another chaperone, whose expression increased in all cases (except for RCh administered as a part of HFCD), is *Dnajb1* (Hsp40), which is functionally related to the previous gene, stimulating the ATPase activity of Hsp70, preventing the aggregation of misfolded proteins, inhibiting apoptosis, and increasing the heat resistance of liver cells [59]. Previously, we showed a positive DE of this gene in DBCB mice treated with HFCD [16].

A positive DE response to all applied dietary manipulations was noted in *Hspa1a* (synonyms *Hsp72*; *hsp68*), which is closely functionally related to *Dnajb1* and *Hspa1b*. Due to interaction with membrane anionic phospholipids, the product of this gene regulates the function of lysosomes, apoptosis, and tumor growth [60]. A decrease in *HSP72* expression is observed in a model of type 2 diabetes mellitus in mice [61].

Thus, a comparative analysis of the DE profiles of individual functionally significant genes in DBA/2J mice, relatively resistant to the development of DIO, showed that these genes can be conditionally assigned to the following three groups. Firstly, these are genes that are predominantly functionally associated with the development of an immune response and inflammation in the liver tissue provoked by the consumption of HFCD (e.g., *Ccl24*, *Fabp7*, *Ifi2712a*, *Cd74*, *H2-Ab1*, *H2-DMb1*, *Igfbp6*, *Plk3*, *Serpina3g*, *Dnaic1*, etc.), and an introduction of the RC supplement along with the same diet has a normalizing (compensating) effect on the expression of these genes. Secondly, this is an extensive heterogeneous group of genes, including nuclear transcription factors, enzymes of the CYP450 family, and circadian rhythm proteins, including *Ppard*, *Irs1*, *Tsku*, *Cyp26b1*, *Il1r1*, *Per1*, *Rgs16*, *Nr1d2*, *Onecut1*, *Grem2*, *Klf9*, *Lpin1*, *G6pc*, etc., whose overlapping responses to HFCD and RC with CD are interpreted as compensatory adaptations supporting obesity resistance in DBA/2J mice. Characteristically, in DBA/2J mice, the sign of DE of these genes in response to HFCD was, in some cases, opposite to that observed by us in previous studies [12,13,14,15,16,17,18] in animals genetically predisposed to the development of DIO (hereditarily obese Zucker rats, male and female Wistar rats, and DBCB tetrahybrid mice). This suggests that in animals resistant to the development of DIO, such as DBA/2J mice used by us, there are compensatory mechanisms excited by excess fat and fructose in the diet, leading to increased energy expenditure and capable of absorbing adverse effects of excess calories on metabolism in the same way as is observed under the influence of dietary supplements with hypolipidemic and anti-obesogenic properties. The ability of animals to show such a compensatory response to the consumption of a hypercaloric diet is apparently associated with the expression features determined by the polymorphism of the genes encoding key regulators of carbohydrate-energy and lipid metabolism. Therefore, within the framework of this study, the third group should include candidate key regulators such as the PPAR family genes, as well as *Srebf1* and, especially, *Socs2*. It makes sense to pay attention to the possible presence of polymorphisms of these genes when translating the results obtained in in vivo models into the practice of clinical personalized diet therapy for patients with obesity and metabolic syndrome.

The link that carries out the transition from the transcriptomic profile of organs, tissues, and the body as a whole to its phenotypic features is metabolic pathways. Thus, in the present study, it was shown that the nature of changes in the metabolic pathway of Retinol metabolism only partially corresponded to that observed under the action of l-Car in DBCB mice prone to DIO [16]. This metabolic pathway was identified as a target for the effects of HFCD, l-Car, and Res in studies [16,17], as well as in our study on Wistar rats treated with HFCD and RC [18]. Metabolic pathways that have been altered in DBA/2J mice by HFCD include mmu00100 (Steroid biosynthesis), mmu00590 (Arachidonic acid metabolism), mmu00071 (Fatty acid metabolism), mmu00591 (Linoleic acid metabolism), and mmu04630 (Jak-STAT signaling pathway), which coincide with our previous results obtained in alternative mouse [16] and rat [17] models of hyperlipidemia, diet-induced obesity, and metabolic syndrome. Thus, the most significant effector links of metabolism are revealed, which are involved in the development of disorders caused by an excessive energy value of the diet, including the metabolic pathways of hydroxy derivatives of fatty acids (oxylipins and prostanoids), steroid hormones (including glucocorticoids), retinoic acid derivatives, and, specifically in a mouse model, Jak/STAT signaling-mediated immune responses. As a link that combines various metabolic reactions into a single-response profile to a hypercaloric diet and biologically active substances, one should point to the PPAR signaling pathway, which demonstrates multidirectional changes in the expression of lipid metabolism genes, including activation of lipogenesis via SCD-1 (stearyl-CoA desaturase and D6 desaturase) and ME (malic enzyme) and peroxisomal fatty acid oxidation under the influence of both HFCD and RC supplement. Consumption of RC on the background of HFCD led to an improvement in PPAR signaling, restoration of retinoid metabolism, and immune-related pathways. It is quite certain that RC does not act by isolated transcription changes, but rather by coordinated recovery of lipid metabolism, retinoid, and hepatic immune homeostasis. These pathway-level changes were consistent with the histological improvement observed in the liver, where RC attenuated HFCD-associated steatotic and inflammatory alterations. The PPAR-related changes are consistent with resveratrol-dependent activation of the PPARα axis and with L-carnitine-associated suppression of hepatic lipid accumulation, oxidative stress, and fibrosis in MASLD models [62,63]. Retinoid-related changes are as well biologically plausible because retinoic acid improves insulin sensitivity in mice with MASLD induced by HFCD/HFD and increases energy expenditure, adipose fatty-acid oxidation, and thermogenic gene expression [64,65]. The activation of immune genes and genes responsible for antigen presentation is consistent with the known ability of retinoic acid to modulate liver immune responses, indicating that RC can influence both the metabolic and inflammatory components of liver stress caused by the HFCD diet [66,67]. Although bioinformatic analysis identified significant alterations in main metabolic pathways, the interpretation of these data should account for potential post-transcriptional and post-translational regulatory mechanisms that may affect the final metabolic endpoints. Thus, the observed transcriptomic response serves as an important indicator of the involvement of specific metabolic pathways; however, further proteomic and metabolomic studies are needed to establish direct functional consequences.

### Limitations of the Study

This study has a number of limitations that should be considered when interpreting the results. The use of male mice only limits the generalizability of findings to females, and future studies should examine sex-specific responses to RC supplementation. The study employed a single mouse strain DBA/2J; more comparative studies across multiple strains would be valuable to distinguish strain-specific from universal responses. The transcriptomic analysis was performed on a relatively small sample size (*n* = 4 per group), which, while consistent with standard microarray practice, may limit the detection of small effect sizes. We used microarray technology rather than RNA-sequencing; while microarrays provide reliable and reproducible data for known transcripts, they do not capture novel transcripts or splice variants. qPCR validation of key differentially expressed genes was not performed; this should be addressed in future studies. Protein-level validation was not conducted, and the functional consequences of transcriptomic changes at the protein level remain to be determined. Histological assessment was qualitative and designed as an additional morphological assessment, not as a quantitative endpoint; the quantitative scoring systems or specialized lipid stains (Oil Red O, BODIPY) would provide additional rigor. The present study should be viewed as an exploratory analysis of transcriptomic responses to HFCD in mice, designed to generate mechanistic hypotheses rather than to infer immediate personalized therapeutic recommendations.

## 4. Materials and Methods

### 4.1. Design of the Experiment

**Figure 10:**
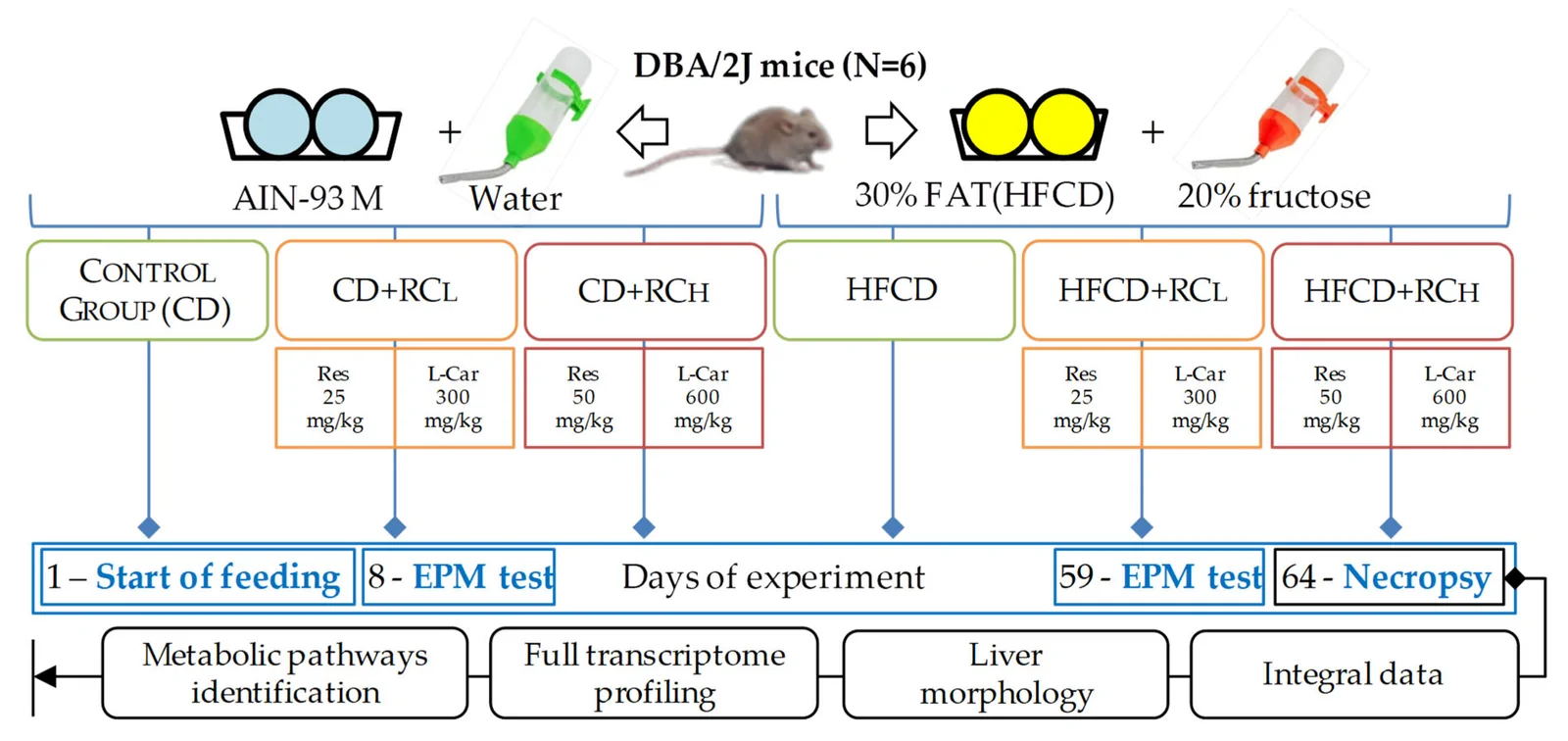
Study design.

We used six groups of six male mice of the inbred line DBA/2J, obtained at the age of 6 weeks from the nursery of the Stolbovaya branch of the Federal State Budgetary Institution of Science “Scientific Center for Biomedical Technologies of the Federal Medical and Biological Agency of Russia” with an initial body weight of 23 ± 2 g. Work with animals was performed in accordance with international recommendations [68]. The design of the experiment was approved by the Ethics Committee of the Federal Research Center of Nutrition and Biotechnology (Protocol No. 4 of 20 April 2017). After 1-week quarantine, mice of the control group (group 1) received a standard semi-synthetic balanced diet (hereinafter referred to as CD) according to AIN93M with minor modifications [69] and drinking water purified by reverse osmosis. Mice of group 2 received CD and a complex dietary supplement, a source of Res and l-Car (hereinafter referred to as RC), at the calculated daily doses of two components equal to 25 and 300 mg/kg of body weight (BW), respectively (low dose of the supplement, hereinafter referred to as RCl). Mice of group 3 received CD and RC at calculated doses of 50 and 600 mg/kg BW, based on its two components, respectively (high dose of the supplement, hereinafter referred to as RCh). Animals of group 4 were fed a high-fat, high-carbohydrate diet (hereinafter referred to as HFCD). The HFCD was a modified AIN-93M diet (identical to CD in all components except carbohydrate and fat content). For HFCD, we replaced a portion of the corn starch with a 1:1 mixture of refined sunflower oil and pork lard to achieve 30% fat by dry weight, and drinking water was replaced with a 20% fructose solution. Animals of group 5 were fed HFCD with RCl; those in group 6 were fed HFCD with RCh. The composition of the experimental diets is presented in Supplementary Table S1. The duration of the experiment was 64 days. The design of the experiment is shown in Figure 10.

The doses of Res (25 and 50 mg/kg BW) and l-Car (300 and 600 mg/kg BW) were selected based on our previous dose-response studies in rats and mice, which demonstrated significant metabolic effects at these levels [16,17,18]. The components of the complex dietary supplement were added directly to the diets. We used Res produced by DSM (Kaiseraugst, Switzerland), trademark resVida^®^, 98% purity according to HPLC, and l-Car manufactured by WIRUD (Bad Homburg, Germany), >98% purity according to HPLC. To maintain the stability of the doses of BACs administered to animals, their specific content in the feed was adjusted daily in accordance with the consumed amount.

Mice were kept by two individuals in polycarbonate cages at a temperature of 21 ± 1 °C and with artificial lighting in the mode of alternating light and dark for periods of 12 h. The amount of food and liquid consumed, as well as the appearance of the animals, were monitored daily; body weight was determined once a week with an accuracy of ±1 g on an electronic balance. On the sixty-fourth day from the start of feeding, mice were taken out of the experiment by decapitation under ether anesthesia, and the mass of internal organs, retroperitoneal white and brown adipose tissue, was determined on electronic balance with an accuracy of ±10 mg. Two samples of liver tissue were taken under aseptic conditions. The first sample was immediately fixed in a 3.7% formaldehyde solution in 0.1 M sodium phosphate buffer pH 7.00, after which alcohol dehydration and embedding in paraffin blocks were performed; 5 μm thick sections were made on a microtome and stained with hematoxylin-eosin according to standard methods and viewed using AxioVision Rel.4.8 software (Carl Zeiss) in an AxioImager Zl microscope (manufactured by Carl Zeiss, Oberkochen, Baden-Wuttemberg, Germany) equipped with a digital camera at a magnification of ×400. Histological examination was performed qualitatively to provide visual morphological support for the transcriptomic and phenotypic data. The second sample of liver tissue was immediately frozen and stored at −80 °C until the start of the transcriptome study. Four liver samples from each group of animals selected randomly were used for transcriptome studies in accordance with the number of sample locations (24 pcs) on three used DNA microarrays (three slides, eight samples per slide).

### 4.2. Behavioral Reactions Examination

Behavioral reactions of mice (anxiety-like functions, levels of mobility, locomotor, and search activity) were studied in the physiological EPM test according to the method described earlier [69]. The study was carried out twice, on the eighth and fifty-ninth days of the experiment.

### 4.3. Preparation of Total RNA and Full Transcriptome Profiling

Total RNA was isolated from each liver sample using the Agilent Total RNA Isolation Mini Kit (Agilent Technologies, Santa Clara, CA, USA) according to the method presented in [13]. Full transcriptome profiling of the liver on microarrays included in the Gene Expression Hybridization Kit (Agilent Technologies, Santa Clara, CA, USA) was performed using the Agilent One-Color Microarray-Based Gene Expression Analysis Low Input Quick Amp Labeling protocol, version 6.8. We used SurePrint G3 Mice Gene Expression v2 8x60K Microarray Kit microchips (catalog number G4852B, Agilent Technologies, Santa Clara, CA, USA). According to the manufacturer’s data, the microarray contains 60 base-long oligonucleotide probes that cover 27,122 genes of the domestic mouse (*Mus domesticus*), 4578 long intergenic noncoding RNAs (lincRNAs) (https://www.agilent.com/en/product/gene-expression-microarray-platform/gene-expression-exon-microarrays/model-organism-microarrays/sureprint-g3-mouse-gene-expression-microarrays-228472#zoomELIBRARY_669002, accessed on 31 August 2026). A total of 24 independent samples of liver RNA, four samples from each group of animals, were analyzed on three microarrays. Microchip scanning was performed on a SureScan Microarray Scanner manufactured by Agilent Technologies. The magnitude of differential expression (DE) was expressed as the base-two logarithm (log2FC) of the increase or decrease ratio in fluorescence compared to the group of comparison after normalization to internal microarray controls (Spike-In). The raw and processed microarray data have been deposited in the Gene Expression Omnibus (GEO) database under accession number GSE344237. The following comparisons to evaluate the gene expression changes by groups of animals were used:

(1) those who received HFCD compared with those who received CD (hereinafter referred to as HFCD/CD).
(2) those who received RCl with HFCD compared with HFCD (RCl + HFCD/HFCD).
(3) those who received RCh with HFCD compared with HFCD (RCh + HFCD/HFCD).
(4) those who received RCl with CD compared with CD (RCl + CD/CD).
(5) those who received RCh with CD compared with CD (RCh + CD/CD).

Data on DE were taken into account if more than a 1.41-fold change was noted in the magnitude of gene expression in the direction of both increase and decrease (|log2FC| ≥ 0.5 with an adjusted *p*-value < 0.05).

### 4.4. Bioinformatical and Statistical Analysis

Microarray scan data were loaded into the “R” environment v4.5.3, and bioinformatics analysis was performed with quantile normalization and further DE analysis in the limma package. Metabolic pathways that are targets of the applied dietary influences were identified using the international database of genes, metabolic pathways, and functions of biological systems, Kyoto Encyclopedia of Genes and Genomes (KEGG) [70]. AnnotationDbi v1.70.0, org.Mm.eg.db v3.20, pathview v1.53.0, gage v3.23, gageData packages V.13, and standard “R” graphics were used to visualize the results of the analysis, completed with additional packages ggplot2 v4.0.3, ggrepel v0.9.8, and gplots v3.3.0. The significance of expression changes was assessed by analyzing the logarithms of fluorescence intensity normalized to internal control (Spike-In) using the Benjamini–Hochberg multiple correction *t*-test [71]. Mean (*M*) and standard error of the mean (*s.e.m*.), Pearson’s correlation coefficients (*r*) and their reliability (α), and significance (*p*) of differences according to the Mann–Whitney test were calculated using the SPSS 20.0 software package.

## 5. Conclusions

Thus, the current studies have shown that in DBA/2J male mice, genetically resistant to the development of DIO, the consumption of an HFCD with an increased specific energy value leads to changes in the transcriptome of liver cells, which, in a large number of cases, extend to the same genes and molecular markers, which were targets of exposure to HFCD in rodents of other lines that are more prone to the formation of the DIO phenotype, but, at the same time, have an opposite direction compared to them and, in some cases, coincide with the effect of feeding with a complex dietary supplement, which apparently is able to exert hypolipidemic and anti-obesogenic action. Notably, RC modulated liver transcriptomic responses in a diet- and dose-dependent manner. Despite the resistance of DBA/2J mice to body weight gain on HFCD, they retained clear evidence of metabolic alterations, including increased white adipose tissue mass and hepatic fat accumulation, indicating that resistance to weight gain does not equate to resistance to metabolic dysfunction. These findings in total suggest the presence of a specific mechanism, triggered by excessive consumption of dietary fat and fructose, that results in absorbing the adverse effects in DIO-resistant animals. This mechanism may be based on the presence of polymorphisms of key genes that regulate carbohydrate-energy and lipid metabolism, of which, according to the present study, the genes of the PPAR family, *Srebf1* and *Socs2*, are of greatest interest. Although the findings require further validation at the transcriptional and protein levels, our results provide a useful transcriptomic framework for understanding RC effects in the context of obesity resistance. In the future, when translating these studies into the practice of personalized diet therapy, the assessment of the polymorphism of these genes deserves special attention. The pathways of steroid hormone biosynthesis, the exchange of PUFA oxy-derivatives, and the Jak-STAT signaling pathway, the role of which was previously revealed in alternative in vivo models, were confirmed as effector links of metabolism activated by the consumption of HFCD. Pathways of PPAR signaling, presentation of immune system antigens, and metabolism of retinol derivatives were identified as metabolic pathways that were simultaneously targets of a complex dietary supplement with l-Car and Res as well as HFCD in DBA/2J mice, which is also consistent with the data of previous studies [16,17] performed on animals fed with l-Car and Res separately. This can be considered as a manifestation of the non-random nature and reproducibility of the data of transcriptomic studies, which makes it possible to identify molecular markers of metabolic changes caused by the development of obesity and exposure to biologically active substances, with the prospect of translating these markers into the practice of clinical diagnostics if suitable biosubstrates are available for this.

## Figures and Tables

**Table 1:** The list of genes in the liver of DBA/2J mice that responded with differential expression to consumption of food supplement containing trans-resveratrol and l-carnitine (RC) at low (RCl) and high (RCh) doses, depending on feeding with basal control diet (CD) or high-fat, high-carbohydrate diet (HFCD).

Group of Genes, Response	log_2_FC > 0.5	log_2_FC < −0.5
Only to RCl as a part of CD	*Arl9*; C2cd4d *; **Cd207** *; E2f2 *; *Etohd2*; *Foxj2*; *Il17d*; Mid1ip1 *; *Nab2*; *Olfr128*; *Olfr1444*; ***Pnpla5*** *; Proser2 *; ***Prox1***; *Rnf43*; *Smim22*; ***Smoc2*** *; *Tent5a*; *Tpgs1*	Abhd17b *; **Apoa4** *; Arid5b *; Chrna2 *; **Cyp2c40** *; **Cyp2c67** *; **Cyp2c68** *; **Cyp2c69** *; Depp1 *; *Fam193b*; *Ifit1*; *Isg20*; *Kdm5b*; *Leng9*; *Lifr*; *Nr0b2*; Oas2 *; *Pik3r1*; *Ppan*; *Pprc1*; *Resf1*; *Sdf2l1*; *Stap1*; *Trp53inp2*; ***Tuft1***; *Zfp809*
Only to RCh as a part of CD	***Acnat2*** *; *Agtr1a*; ***Arhgap10*** *; *Atf4*; *Atp7b*; Blcap *; *Bok*; *Btg1*; *Cables1*; ***Celsr1*** *; ***Cldn1***; *Cmpk2*; ***Coq8a*** *; *Cpeb4*; *Crip2*; Cyp2b9 *; Cyp4b1 *; *Dapk2*; Ddc *; *Desi2*; ***Dnmt3b***; *Efnb1*; *Enpp3*; *Eps8l2*; *Erc2*; ***Fam160a1***; *Flvcr2*; *Fpgs*; ***G6pc*** *; ***Gclc***; *Gdf15*; Gldc *; *Glt28d2*; *Gtf2ird1*; *Hacl1*; *Hdac5*; ***Hhex***; *Hspbap1*; ***Id2***; ***Insig2*** *; *Irf6*; *Klf2*; ***Leap2*** *; **Lgalsl** *; *Lipa*; *Lyz1*; *Mcu*; ***Mreg***; ***Mrnip***; *Nectin1*; *Nedd4l*; Net1 *; Nfe2 *; **Nr5a1** *; Olfr398 *; ***Osgin1***; *Phf13*; *Pid1*; *Pik3ca*; Pltp *; Pnkd *; Pnrc1 *; *Prelid2*; Pter *; *Pxdc1*; *Pxmp4*; ***Rnf125*** *; *Rxrg*; *Sco2*; *Serpina7*; Sigmar1 *; *Slc16a1*; *Slc19a2*; *Smagp*; *Snora81*; *Sntb2*; *Spata13*; *Sphk2*; *Spsb2*; *Stat5a*; *Stat5b*; *Syne1*; ***Tab2*** *; *Taok3*; Tars *; **Tjp3** *; **Trim2** *; **Tubb2a-ps2** *; *Tymp*; *Vmn1r48*; *Wdr91*; *Wrnip1*; Xntrpc *; *Ywhae*	*Aifm2*; Alpl *; *Azin1*; *Chordc1*; ***Coq10b*** *; *Cry2*; *Ctps*; ***Cyp7a1***; ***Dclre1a*** *; **Dnajb11** *; *Dtymk*; ***Efhd2*** *; *Egfr*; *Epop*; ***Etnk2***; ***Fabp4***; *Fam214a*; Fam25c *; ***Fam47e***; Gbp11 *; **Gdap10** *; *Gde1*; *Gin1*; *Gnl3*; Gpcpd1 *; Gys2 *; *Hbb-b1*; *Hes6*; *Homer2*; Hsd17b6 *; **Il12rb1** *; Il15ra *; *Il1rap*; *Ing3*; *Ints2*; *Ip6k2*; Klhl21 *; *Lonrf1*; Lpin2 *; **Maff** *; *Mbnl2*; Nampt *; **Narf** *; *Odc1*; *P2ry1*; *Pck1*; *Pde5a*; *Per2*; *Pex26*; *Pfkfb1*; *Pfkfb2*; ***Prr5l***; ***Rapgef4*** *; **Rhbdd2** *; Rrp9 *; Sds *; *Sec23b*; Slc16a12 *; *Slc37a4*; Snx10 *; ***Srm***; Tha1 *; Trmt61a *; *Txndc11*; *Zfp330*; *Zfp445*; *Zfp967*
To both doses of RC as a part of CD only	*Atoh8*; *Cnksr3*; ***Cyr61*** *; Ddit4 *; *Fam89a*; *Frat1*; *Gata6*; *Id3*; Ihh *; *Irs1*; ***Krt6a*** *; ***Notch1***; Pdgfc *; Ppard *; **Ppp1r3c** *; *Rhpn2*; Rnps1 *; Rsrp1 *; **Sertad3** *; Slc13a2 *; Spsb4 *; *Stra6*; ***Tbx3*** *; *Tnfaip2*; Zfp36l3 *	Aacs *; **Alas1** *; Angptl4 *; Arfgap3 *; **Arhgef26** *; *Banp*; Ccdc134 *; ***Creld2***; Eif4ebp3 *; Etfbkmt *; Fkbp5 *; *Gmppb*; *Golph3l*; ***Grem2*** *; *Herpud1*; Klf9 *; Lpin1 *; Mafb *; *Mbd1*; Mcm10 *; Mtss1 *; *Nol8*; Nr1d1 *; Nr1d2 *; **Nrep** *; **Onecut1** *; *Phf8*; ***Pim1*** *; Pim3 *; Plcxd2 *; *Rgs5*; Rpp38 *; **Serpina4-ps1** *; *Serpina9*; Slc20a1 *; ***Syvn1***; Tsku *; Xbp1 *
Only to RCl as a part of HFCD	***Bag3*** *; *Cxcl1*; *Eif1a*; *Hspa8*; Serpina3f *; *Stab2*; Zfp36l2 *;	*Erf*; *Hrasls5*; ***Lrtm1*** *; **Ntrk2** *; ***Phka2***; *Plekha6*; *Plekhg5*; *Plekhg6*; *Rnf152*; Scd1 *; *Sipa1l2*; *Spata2l*; Trib3 *
Only to RCh as a part of HFCD	Acmsd *; *Aldh7a1*; *Aox3*; Arhgap24 *; *Capg*; ***Ccl24*** *; ***Ccl5***; ***Ccnd1*** *; Cdh1 *; *Clec2d*; Clec7a *; Coro1a *; *Cryaa*; Cyp3a11 *; Cyp3a16 *; **Fabp7** *; *Foxa2*; Gbp2 *; Gbp3 *; **Gbp6** *; **Gsta2** *; *Gsta3*; ***Gzma***; ***H2-Eb1*** *; *Hist1h2ab*; *Hist1h2ao*; *Hmgb1*; *Hsp90aa1*; Ifi202b *; Ifi27l2a *; ***Igfbp2***; Irgm2 *; Itgb7 *; *Lgals3*; ***Lpl***; Ltb *; **Ly6a** *; *Ly6c1*; *Ly6c2*; Mgp *; **Moxd1** *; Myo1g *; *Olfr6*; *Olfr960*; Phf11b *; ***Rhox2e***; *Rs1*; *Rsg1*; *Rtp4*; *S100a4*; S100a6 *; **Sftpd** *; Stmn1 *; Sucnr1 *; *Tatdn2*; Usp18 *; *Wdfy1*; *Zfp157*	*Adam11*; ***Apcs***; ***B3galt1*** *; *Bmi1*; *Ceacam1*; ***Cxcl14***; *Cyp2d40*; *Cyp3a13*; *Efna1*; ***Fgl1***; *Fkbp1a*; *Fscn2*; *Gas6*; *Gpc1*; *Gprc5a*; *Gstp3*; *Gtf2a2*; *Il27*; ***Isyna1*** *; *Itih3*; *Itih4*; *Khk*; *Krtap11-1*; *Lbp*; ***Lcn2*** *; *Lpgat1*; *Lrg1*; *Lrig1*; *Mat1a*; ***Mt1*** *; ***Mta1***; *Orm1*; ***Orm2***; P2ry2 *; *Pklr*; *Prg4*; *Rpap3*; *Serpina10*; ***Serpina3n***; *Slc13a3*; *Steap4*; *Zbtb48*; *Zfp385c*
To both doses of RC as a part of HFCD only	*Bcl2a1d*; *Ccnb2*; Cd74 *; ***Cxcl10***; *Cxcl9*; Dnaic1 *; **Egr1** *; *Ehhadh*; *Foxa1*; Gvin1 *; H2-Ab1 *; H2-DMb1 *; *Hspb1*; *Hsph1*; Igfbp6 *; **Plk3** *; Serpina3g *; *Sgsm1*; ***Tgtp2*** *	*Bhlhe40*; Cpsf4l *; *Crybb3*; *S1pr5*; ***Saa1***; ***Saa2***; Srebf1 *; *Sult2a7*; *Wipf3*
Only to RCl as a part of both diets ^1^	*Bcl6*; E2f8 *; *Igfbp1*	Dclk38 *
Only to RCh as a part of both diets ^1^	Aqp8 *; Avpr1a *; Birc5 *; *Ces2b*; ***Ces2c***; Clpx *; Dtx4 *; **Elovl3** *; *Ethe1*; *Ifi47*; ***Ndrg1***; *Phlda1*; *Spon2*	*Fam129b*; *G0s2*; ***Ly6d*** *; Noct *; ***Nrg4***; Por *; *Saa4*; *Sbk1*; *Tspan4*
To RCl with CD and to both doses of RC with HFCD ^1^	Hspa1b *	Mt2 *; Orm3 *; Nnmt *; *Kcnk5*
To RCh with CD and to both doses of RC with HFCD ^1^	Acot1 *; Cyp4a10 *; Cyp4a31 *; **Gadd45g** *; *Rassf4*; Tubb2a *	***Cyp2a4*** *; Cyp2a5 *; *Gnat1*; Klf13 *; Slc5a6 *; *Stbd1*; Tef *; Thrsp *; *Upp2*; ***Usp2*** *
To both doses of RC with CD and to RCl with HFCD ^1^	***Derl3*** *; Dnajb1 *; *Dnajb9*; *Ier5*	Hamp2 *
To both doses of RC with CD and to RCh with HFCD ^1^	Cib3 *; Chka *; Cyp26b1 *; *Pou4f1*	Cgref1 *; Dnajc12 *; **Il1r1** *; Rgs16 *; *Scara5*; St3gal5 *
To RCl plus HFCD and to RCh plus CD ^1^	***Fgf21*** *; *Jun*; Nfil3 *; *Zbtb16*	Arl4d *; **Ccne1** *; *Ppargc1b*; *Snai2*
To RCh plus HFCD and to RCl plus CD ^1^	*Amer3*; *Cdh16*; ***Hmox1***; *Olfr1384*; Rgs3 *	*Cyb561*; Inhbe *; ***Saa3***
To both doses of RC as a part of both diets ^1^	Abtb2 *; **Chac1** *; Cish *; **Hspa1a** *; Lrfn3 *; Marco *; Socs2 *; Tff3 *	***Dbp*** *; Hamp *; *Mfsd2a*; Pcsk4 *; Per1 *

Notes: Bold font marks genes in the first quartile of |log_2_FC| value. * genes that responded with DE also to feeding with HFCD, compared with CD. ^1^ the sign of log_2_FC relates to the response of its DE to supplement as a part of HFCD.

**Table 2:** List of metabolic pathways (KEGGs) identified as targets of complex supplement with resveratrol and l-carnitine (RC) in mice fed with a control balanced diet (CD) or high-fat, high-carbohydrate diet (HFCD).

KEGG	Pathway	Diet Fed, Supplement				
		HFCD	RCl+ HFCD	RCh+ HFCD	RCl+ CD	RCh+ CD
mmu00330	Arginine and proline metabolism	0.005	–	–	–	–
mmu00100	Steroid biosynthesis	0.002	–	–	–	–
mmu00260	Glycine, serine, and threonine metabolism	0.023	–	–	–	–
mmu00590	Arachidonic acid metabolism	0.003	–	–	–	–
mmu00071	Fatty acid metabolism	0.025	–	–	–	–
mmu00591	Linoleic acid metabolism	0.036	–	–	–	–
mmu00900	Terpenoid backbone biosynthesis	0.001	–	–	–	–
mmu00380	Tryptophan metabolism	0.030	–	–	–	–
mmu00250	Alanine, aspartate, and glutamate metabolism	0.045	–	–	–	–
mmu00270	Cysteine and methionine metabolism	0.020	–	–	–	–
mmu00340	Histidine metabolism	0.003	–	–	–	–
mmu00350	Tyrosine metabolism	0.007	–	–	–	–
mmu00650	Butanoate metabolism	0.035	–	–	–	–
mmu00280	Valine, leucine, and isoleucine degradation	0.011	–	–	–	–
mmu04975	Fat digestion and absorption	0.043	–	–	–	–
mmu01040	Biosynthesis of unsaturated fatty acids	0.015	–	–	–	–
mmu00360	Phenylalanine metabolism	0.039	–	–	–	–
mmu04630	Jak-STAT signaling pathway	0.015	–	0.010	–	–
mmu03320	PPAR signaling pathway	0.005	0.003	–	–	0.004
mmu00982	Drug metabolism–cytochrome P450	<0.001	–	0.002	–	–
mmu00980	Metabolism of xenobiotics by cytochrome P450	<0.001	–	0.003	–	–
mmu00480	Glutathione metabolism	0.003	–	0.015	–	–
mmu04672	Intestinal immune network for IgA production	0.011	–	0.009	–	–
mmu04145	Phagosome	0.014	–	0.016	–	–
mmu00140	Steroid hormone biosynthesis	0.033	–	0.037	–	–
mmu04612	Antigen processing and presentation	0.016	0.028	0.009	–	–
mmu04010	MAPK signaling pathway	–	0.011	–	–	–
mmu04974	Protein digestion and absorption	–	0.042	–	–	–
mmu04620	Toll-like receptor signaling pathway	–	0.043	–	–	–
mmu04710	Circadian rhythm–mammal	–	0.046	–	–	–
mmu04514	Cell adhesion molecules (CAMs)	–	–	0.008	–	–
mmu04740	Olfactory transduction	–	–	0.042	–	–
mmu00983	Drug metabolism–other enzymes	–	–	0.044	–	–
mmu00830	Retinol metabolism	<0.001	0.027	0.002	–	0.036

## Data Availability

The microarray data generated in this study have been deposited in the GEO database under accession number GSE344237. Other data available on request due to restrictions, e.g., privacy or ethical.

## Supplementary Materials

The following supporting information can be downloaded at: https://www.mdpi.com/article/10.3390/ijms27177808/s1.

## References

[1] Tutelyan V.A., Kiseleva T.L., Kochetkova A.A., Smirnova E.A., Kiseleva M.A., Sarkisyan V.A. (2016). Promising Source of Micronutrients for Specialized Foods with Modified Carbohydrate Profile: Traditional Medicine Experience. Probl. Nutr..

[2] Sun N.N., Wu T.Y., Chau C.F. (2016). Natural Dietary and Herbal Products in Anti-Obesity Treatment. Molecules.

[3] Ríos-Hoyo A., Gutiérrez-Salmeán G. (2016). New Dietary Supplements for Obesity: What We Currently Know. Curr. Obes. Rep..

[4] Panchal S.K., Poudyal H., Ward L.C., Waanders J., Brown L. (2015). Modulation of Tissue Fatty Acids by L-Carnitine Attenuates Metabolic Syndrome in Diet-Induced Obese Rats. Food Funct..

[5] Borra M.T., Smith B.C., Denu J.M. (2005). Mechanism of Human SIRT1 Activation by Resveratrol. J. Biol. Chem..

[6] Ma S., Feng J., Zhang R., Chen J., Han D., Li X., Yang B., Li X., Fan M., Li C. (2017). SIRT1 Activation by Resveratrol Alleviates Cardiac Dysfunction via Mitochondrial Regulation in Diabetic Cardiomyopathy Mice. Oxidative Med. Cell. Longev..

[7] Lu C., Xing H., Yang L., Chen K., Shu L., Zhao X., Song G. (2021). Resveratrol Ameliorates High-Fat-Diet-Induced Abnormalities in Hepatic Glucose Metabolism in Mice via the AMP-Activated Protein Kinase Pathway. Evid.-Based Complement. Altern. Med..

[8] Du F., Huang R., Lin D., Wang Y., Yang X., Huang X., Zheng B., Chen Z., Huang Y., Wang X. (2021). Resveratrol Improves Liver Steatosis and Insulin Resistance in Non-Alcoholic Fatty Liver Disease in Association with the Gut Microbiota. Front. Microbiol..

[9] Wu J., Li Y., Yu J., Gan Z., Wei W., Wang C., Zhang L., Wang T., Zhong X. (2020). Resveratrol Attenuates High-Fat Diet Induced Hepatic Lipid Homeostasis Disorder and Decreases m(6)A RNA Methylation. Front. Pharmacol..

[10] Gao X., Sun C., Zhang Y., Hu S., Li D. (2022). Dietary Supplementation of L-Carnitine Ameliorates Metabolic Syndrome Independent of Trimethylamine N-Oxide Produced by Gut Microbes in High-Fat Diet-Induced Obese Mice. Food Funct..

[11] Zhang Y., Fu Y., Jiang T., Liu B., Sun H., Zhang Y., Fan B., Li X., Qin X., Zheng Q. (2021). Enhancing Fatty Acids Oxidation via L-Carnitine Attenuates Obesity-Related Atrial Fibrillation and Structural Remodeling by Activating AMPK Signaling and Alleviating Cardiac Lipotoxicity. Front. Pharmacol..

[12] Trusov N.V., Apryatin S.A., Gorbachev A.Y., Naumov V.A., Mzhelskaya K.V., Gmoshinski I.V. (2018). The Effect of Hypercaloric Diet and Quercetin on the Full-Transcriptome Liver Tissue Profile of Zucker-LEPRfa Rats. Probl. Endokrinol..

[13] Apryatin S.A., Trusov N.V., Gorbachev A.J., Naumov V.A., Mzhel’skaya K.V., Balakina A.S., Gmoshinski I.V. (2019). Full Transcriptome Profiling of the Liver of Fat-, Fructose and Cholesterol-Fed C57BL/6J Mice. Russ. J. Genet..

[14] Apryatin S.A., Trusov N.V., Gorbachev A.Y., Naumov V.A., Balakina A.S., Mzhel’skaya K.V., Gmoshinski I.V. (2019). Comparative Whole Transcriptome Profiling of Liver Tissue from Wistar Rats Fed with Diets Containing Different Amounts of Fat, Fructose, and Cholesterol. Biochemistry.

[15] Trusov N.V., Apryatin S.A., Shipelin V.A., Gmoshinski I.V. (2020). Full Transcriptome Analysis of Gene Expression in Liver of Mice in a Comparative Study of Quercetin Efficiency on Two Obesity Models. Probl. Endokrinol..

[16] Trusov N.V., Apryatin S.A., Timonin A.N., Shipelin V.A., Gmoshinski I.V., Nikityuk D.B. (2021). Comparative Evaluation of the Effect of Resveratrol and Carnitine on the Full Transcriptomic Profile of Liver Tissue in Mice with Different Sensitivity to the Development of Alimentary Obesity. Tomsk State Univ. J. Biol..

[17] Trusov N.V., Apryatin S.A., Shipelin V.A., Shumakova A.A., Gmoshinski I.V., Nikityuk D.B., Tutelyan V.A. (2021). Effect of Administration of Carnitine, Resveratrol, and Aromatic Amino Acids with High-Fat-High-Fructose Diet on Gene Expression in Liver of Rats: Full Transcriptome Analysis. Russ. J. Genet..

[18] Trusov N.V., Shipelin V.A., Gmoshinski I.V. (2025). Full Transcriptome Analysis of the Liver of Rats Receiving the Complex of L-Carnitine and Resveratrol in the Composition of a Standard or Hypercaloric Diet. Biochem. Mosc. Suppl. Ser. B..

[19] Hagarty-Waite K.A., Totten M.S., Pierce M., Armah S.M., Erikson K.M. (2022). Influence of Sex and Strain on Hepatic and Adipose Tissue Trace Element Concentrations and Gene Expression in C57BL/6J and DBA/2J High Fat Diet Models. Int. J. Mol. Sci..

[20] Totten M.S., Wallace C.W., Pierce D.M., Fordahl S.C., Erikson K.M. (2022). The Impact of a High-Fat Diet on Physical Activity and Dopamine Neurochemistry in the Striatum Is Sex and Strain Dependent in C57BL/6J and DBA/2J Mice. Nutr. Neurosci..

[21] Sweeney P., O’Hara K., Xu Z., Yang Y. (2017). HFD-Induced Energy States-Dependent Bidirectional Control of Anxiety Levels in Mice. Int. J. Obes..

[22] Lee S.H., Chaves M.M., Kamenyeva O., Gazzinelli-Guimaraes P.H., Kang B., Pessenda G., Passelli K., Tacchini-Cottier F., Kabat J., Jacobsen E.A. (2020). M2-Like, Dermal Macrophages Are Maintained via IL-4/CCL24-Mediated Cooperative Interaction with Eosinophils in Cutaneous Leishmaniasis. Sci. Immunol..

[23] Ben-Yehuda C., Bader R., Puxeddu I., Levi-Schaffer F., Breuer R., Berkman N. (2008). Airway Eosinophil Accumulation and Eotaxin-2/CCL24 Expression Following Allergen Challenge in BALB/c Mice. Exp. Lung Res..

[24] Yasumoto Y., Miyazaki H., Ogata M., Kagawa Y., Yamamoto Y., Islam A., Yamada T., Katagiri H., Owada Y. (2018). Glial Fatty Acid-Binding Protein 7 (FABP7) Regulates Neuronal Leptin Sensitivity in the Hypothalamic Arcuate Nucleus. Mol. Neurobiol..

[25] Ohgaki S., Iida K., Yokoo T., Watanabe K., Kihara R., Suzuki H., Shimano H., Toyoshima H., Yamada N. (2007). Identification of ISG12b as a Putative Interferon-Inducible Adipocytokine Which Is Highly Expressed in White Adipose Tissue. J. Atheroscler. Thromb..

[26] Mishra A., Iyer S., Kesarwani A., Baligar P., Arya S.P., Arindkar S., Kumar M.J., Upadhyay P., Majumdar S.S., Nagarajan P. (2016). Role of Antigen Presenting Cell Invariant Chain in the Development of Hepatic Steatosis in Mouse Model. Exp. Cell Res..

[27] Porsche C.E., Delproposto J.B., Patrick E., Zamarron B.F., Lumeng C.N. (2020). Adipose Tissue Dendritic Cell Signals Are Required to Maintain T Cell Homeostasis and Obesity-Induced Expansion. Mol. Cell. Endocrinol..

[28] Putzer P., Breuer P., Götz W., Gross M., Kübler B., Scharf J.G., Schuller A.G., Hartmann H., Braulke T. (1998). Mouse Insulin-Like Growth Factor Binding Protein-6: Expression, Purification, Characterization and Histochemical Localization. Mol. Cell. Endocrinol..

[29] Zimmerman W.C., Erikson R.L. (2007). Polo-Like Kinase 3 Is Required for Entry into S Phase. Proc. Natl. Acad. Sci. USA.

[30] Bouter S., Rodriguez M., Guigal-Stephan N., Courtade-Gaïani S., Xuereb L., de Montrion C., Croixmarie V., Umbdenstock T., Boursier-Neyret C., Lonchampt M. (2010). Coordinate Transcriptomic and Metabolomic Effects of the Insulin Sensitizer Rosiglitazone on Fundamental Metabolic Pathways in Liver, Soleus Muscle, and Adipose Tissue in Diabetic db/db Mice. PPAR Res..

[31] Jideonwo V., Hou Y., Ahn M., Surendran S., Morral N. (2018). Impact of Silencing Hepatic SREBP-1 on Insulin Signaling. PLoS ONE.

[32] Su S., Tian H., Jia X., Zhu X., Wu J., Zhang Y., Chen Y., Li Z., Zhou Y. (2020). Mechanistic Insights into the Effects of SREBP1c on Hepatic Stellate Cell and Liver Fibrosis. J. Cell. Mol. Med..

[33] Xiaoli A.M., Song Z., Yang F. (2019). Lipogenic SREBP-1a/c Transcription Factors Activate Expression of the Iron Regulator Hepcidin, Revealing Cross-Talk between Lipid and Iron Metabolisms. J. Biol. Chem..

[34] Narkar V.A., Downes M., Yu R.T., Embler E., Wang Y.X., Banayo E., Mihaylova M.M., Nelson M.C., Zou Y., Juguilon H. (2015). Integrative and Systemic Approaches for Evaluating PPARβ/δ (PPARD) Function. Nucl. Recept. Signal..

[35] Serrano-Marco L., Barroso E., El Kochairi I., Palomer X., Michalik L., Wahli W., Vázquez-Carrera M. (2012). The Peroxisome Proliferator-Activated Receptor (PPAR) β/δ Agonist GW501516 Inhibits IL-6-Induced Signal Transducer and Activator of Transcription 3 (STAT3) Activation and Insulin Resistance in Human Liver Cells. Diabetologia.

[36] Man X.F., Hu N., Tan S.W., Tang H.N., Guo Y., Tang C.Y., Liu Y.Q., Tang J., Zhou C.L., Wang F. (2020). Insulin Receptor Substrate-1 Inhibits High-Fat Diet-Induced Obesity by Browning of White Adipose Tissue through miR-503. FASEB J..

[37] Sahin K., Kucuk O., Orhan C., Erten F., Sahin N., Komorowski J.R. (2021). Effects of Supplementing Different Chromium Histidinate Complexes on Glucose and Lipid Metabolism and Related Protein Expressions in Rats Fed a High-Fat Diet. J. Trace Elem. Med. Biol..

[38] Wang Q., Sharma V.P., Shen H., Xiao Y., Zhu Q., Xiong X., Guo L., Jiang L., Ohta K., Li S. (2019). The Hepatokine Tsukushi Gates Energy Expenditure via Brown Fat Sympathetic Innervation. Nat. Metab..

[39] Mouchiroud M., Camiré É., Aldow M., Caron A., Jubinville É., Turcotte L., Kaci I., Beaulieu M.J., Roy C., Labbé S.M. (2019). The Hepatokine Tsukushi Is Released in Response to NAFLD and Impacts Cholesterol Homeostasis. JCI Insight.

[40] Takeuchi H., Yokota A., Ohoka Y., Iwata M. (2011). Cyp26b1 Regulates Retinoic Acid-Dependent Signals in T Cells and Its Expression Is Inhibited by Transforming Growth Factor-β. PLoS ONE.

[41] Chenery A., Burrows K., Antignano F., Underhill T.M., Petkovich M., Zaph C. (2013). The Retinoic Acid-Metabolizing Enzyme Cyp26b1 Regulates CD4 T Cell Differentiation and Function. PLoS ONE.

[42] McGillicuddy F.C., Reynolds C.M., Finucane O., Coleman E., Harford K.A., Grant C., Sergi D., Williams L.M., Mills K.H., Roche H.M. (2013). Long-Term Exposure to a High-Fat Diet Results in the Development of Glucose Intolerance and Insulin Resistance in Interleukin-1 Receptor I-Deficient Mice. Am. J. Physiol. Endocrinol. Metab..

[43] McGillicuddy F.C., Harford K.A., Reynolds C.M., Oliver E., Claessens M., Mills K.H., Roche H.M. (2011). Lack of Interleukin-1 Receptor I (IL-1RI) Protects Mice from High-Fat Diet-Induced Adipose Tissue Inflammation Coincident with Improved Glucose Homeostasis. Diabetes.

[44] Hirota T., Okano T., Kokame K., Shirotani-Ikejima H., Miyata T., Fukada Y. (2002). Glucose Down-Regulates Per1 and Per2 mRNA Levels and Induces Circadian Gene Expression in Cultured Rat-1 Fibroblasts. J. Biol. Chem..

[45] Hayasaka N., Aoki K., Kinoshita S., Yamaguchi S., Wakefield J.K., Tsuji-Kawahara S., Horikawa K., Ikegami H., Wakana S., Murakami T. (2011). Attenuated Food Anticipatory Activity and Abnormal Circadian Locomotor Rhythms in Rgs16 Knockdown Mice. PLoS ONE.

[46] Amador A., Kamenecka T.M., Solt L.A., Burris T.P. (2018). REV-ERBβ Is Required to Maintain Normal Wakefulness and the Wake-Inducing Effect of Dual REV-ERB Agonist SR9009. Biochem. Pharmacol..

[47] Amador A., Wang Y., Banerjee S., Kamenecka T.M., Solt L.A., Burris T.P. (2016). Pharmacological and Genetic Modulation of REV-ERB Activity and Expression Affects Orexigenic Gene Expression. PLoS ONE.

[48] Jacquemin P., Durviaux S.M., Jensen J., Godfraind C., Gradwohl G., Guillemot F., Madsen O.D., Carmeliet P., Dewerchin M., Collen D. (2000). Transcription Factor Hepatocyte Nuclear Factor 6 Regulates Pancreatic Endocrine Cell Differentiation and Controls Expression of the Proendocrine Gene Ngn3. Mol. Cell. Biol..

[49] Tanwar V., Bylund J.B., Hu J., Yan J., Walthall J.M., Mukherjee A., Heaton W.H., Wang W.D., Potet F., Rai M. (2014). Gremlin 2 Promotes Differentiation of Embryonic Stem Cells to Atrial Fate by Activation of the JNK Signaling Pathway. Stem Cells.

[50] Pei H., Yao Y., Yang Y., Liao K., Wu J.R. (2011). Krüppel-Like Factor KLF9 Regulates PPARγ Transactivation at the Middle Stage of Adipogenesis. Cell Death Differ..

[51] Chen S.H., Zhuang X.H., Liu Y.T., Sun A.L., Chen C.R. (2012). Expression and Significance of Lipin1 and AMPKα in Hepatic Insulin Resistance in Diet-Induced Insulin Resistance Rats. Exp. Clin. Endocrinol. Diabetes.

[52] Kim G.-Y., Lee Y.M., Cho J.-H., Pan C.-J., Jun H.S., Springer D.A., Mansfield B.C., Chou J.Y. (2015). Mice Expressing Reduced Levels of Hepatic Glucose-6-Phosphatase-α Activity Do Not Develop Age-Related Insulin Resistance or Obesity. Hum. Mol. Genet..

[53] Dey B.R., Spence S.L., Nissley P., Furlanetto R.W. (1998). Interaction of Human Suppressor of Cytokine Signaling (SOCS)-2 with the Insulin-Like Growth Factor-I Receptor. J. Biol. Chem..

[54] Letellier E., Haan S. (2016). SOCS2: Physiological and Pathological Functions. Front. Biosci. (Elite Ed.).

[55] Kato H., Nomura K., Osabe D., Shinohara S., Mizumori O., Katashima R., Iwasaki S., Nishimura K., Yoshino M., Kobori M. (2006). Association of Single-Nucleotide Polymorphisms in the Suppressor of Cytokine Signaling 2 (SOCS2) Gene with Type 2 Diabetes in the Japanese. Genomics.

[56] Reeg S., Jung T., Castro J.P., Davies K.J.A., Henze A., Grune T. (2016). The Molecular Chaperone Hsp70 Promotes the Proteolytic Removal of Oxidatively Damaged Proteins by the Proteasome. Free Radic. Biol. Med..

[57] Martine P., Chevriaux A., Derangère V., Apetoh L., Garrido C., Ghiringhelli F., Rébé C. (2019). HSP70 Is a Negative Regulator of NLRP3 Inflammasome Activation. Cell Death Dis..

[58] Wolf J.H., Bhatti T.R., Fouraschen S., Chakravorty S., Wang L., Kurian S., Salomon D., Olthoff K.M., Hancock W.W., Levine M.H. (2014). Heat Shock Protein 70 Is Required for Optimal Liver Regeneration after Partial Hepatectomy in Mice. Liver Transplant..

[59] Uchiyama Y., Takeda N., Mori M., Terada K. (2006). Heat Shock Protein 40/DjB1 Is Required for Thermotolerance in Early Phase. J. Biochem..

[60] McCallister C., Kdeiss B., Oliverio R., Nikolaidis N. (2016). Characterization of the Binding between a 70-kDa Heat Shock Protein, HspA1A, and Phosphoinositides. Biochem. Biophys. Res. Commun..

[61] Kitano S., Kondo T., Matsuyama R., Ono K., Goto R., Takaki Y., Hanatani S., Sakaguchi M., Igata M., Kawashima J. (2019). Impact of Hepatic HSP72 on Insulin Signaling. Am. J. Physiol. Endocrinol. Metab..

[62] Huang Y., Lang H., Chen K., Zhang Y., Gao Y., Ran L., Yi L., Mi M., Zhang Q. (2020). Resveratrol Protects against Nonalcoholic Fatty Liver Disease by Improving Lipid Metabolism and Redox Homeostasis via the PPARα Pathway. Appl. Physiol. Nutr. Metab..

[63] Mollica G., Senesi P., Codella R., Vacante F., Montesano A., Luzi L., Terruzzi I. (2020). L-Carnitine Supplementation Attenuates NAFLD Progression and Cardiac Dysfunction in a Mouse Model Fed with Methionine and Choline-Deficient Diet. Dig. Liver Dis..

[64] Tsuchiya H. (2012). Retinoids as promising treatment for non-alcoholic fatty liver disease. Yakugaku Zasshi.

[65] Zhu S., Zhang J., Zhu D., Jiang X., Wei L., Wang W., Chen Y.Q. (2022). Adipose Tissue Plays a Major Role in Retinoic Acid-Mediated Metabolic Homoeostasis. Adipocyte.

[66] Jie Z., Liang Y., Yi P., Tang H., Soong L., Cong Y., Zhang K., Sun J. (2017). Retinoic Acid Regulates Immune Responses by Promoting IL-22 and Modulating S100 Proteins in Viral Hepatitis. J. Immunol..

[67] Baek K.-R., Kim H.-K. (2025). All-Trans Retinoic Acid Attenuates Inflammation and Insulin Resistance Induced by Adipocyte–Macrophage Coculture. Molecules.

[68] European Parliament and Council (2010). Directive 2010/63/EU on the Protection of Animals Used for Scientific Purposes.

[69] Apryatin S.A., Shipelin V.A., Trusov N.V., Mzhelskaya K.V., Evstratova V.S., Kirbaeva N.V., Soto J.S., Fesenko Z.S., Gainetdinov R.R., Gmoshinski I.V. (2019). Comparative Analysis of the Influence of a High-Fat/High-Carbohydrate Diet on the Level of Anxiety and Neuromotor and Cognitive Functions in Wistar and DAT-KO Rats. Physiol. Rep..

[70] Kyoto Encyclopedia of Genes and Genomes (KEGG). https://www.genome.jp/kegg/.

[71] Benjamini Y., Hochberg Y. (1995). Controlling the False Discovery Rate: A Practical and Powerful Approach to Multiple Testing. J. R. Stat. Soc. Ser. B.

